# Climate smart agriculture and global food-crop production

**DOI:** 10.1371/journal.pone.0231764

**Published:** 2020-04-29

**Authors:** Alessandro De Pinto, Nicola Cenacchi, Ho-Young Kwon, Jawoo Koo, Shahnila Dunston

**Affiliations:** 1 Environment and Production Technology Division, International Food Policy Research Institute, Washington, DC, United States of America; 2 Energy Systems Division, Argonne National Laboratories, Lemont, IL, United States of America; University College London, UNITED KINGDOM

## Abstract

Most business-as-usual scenarios for farming under changing climate regimes project that the agriculture sector will be significantly impacted from increased temperatures and shifting precipitation patterns. Perhaps ironically, agricultural production contributes substantially to the problem with yearly greenhouse gas (GHG) emissions of about 11% of total anthropogenic GHG emissions, not including land use change. It is partly because of this tension that Climate Smart Agriculture (CSA) has attracted interest given its promise to increase agricultural productivity under a changing climate while reducing emissions. Considerable resources have been mobilized to promote CSA globally even though the potential effects of its widespread adoption have not yet been studied. Here we show that a subset of agronomic practices that are often included under the rubric of CSA can contribute to increasing agricultural production under unfavorable climate regimes while contributing to the reduction of GHG. However, for CSA to make a significant impact important investments and coordination are required and its principles must be implemented widely across the entire sector.

## 1 Introduction

Uncertainty in projections makes it difficult to determine the precise impact of climate change on future agricultural productivity, but studies have consistently found that under most scenarios significant negative effects should be expected worldwide [1–6] and especially in economically underdeveloped regions [7–9]. Importantly, agricultural production is not only affected by climate change but contributes substantially to the problem with yearly greenhouse gas (GHG) emissions that range from 5.0 to 5.8 Gt CO_2_ e or about 11% of total anthropogenic GHG emissions [10]. Combined with forestry and other land uses, anthropogenic land activities contribute about a quarter of annual GHG emissions, which is the equivalent of 10 to 12 Gt CO_2_ e per year [10].

Recent developments in the United Nations Framework Convention on Climate Change negotiations (i.e. the Paris Agreement in 2015 and the Koronivia joint work on agriculture [11,12]) and the recent Intergovernmental Panel on Climate Change special report [13] have reinvigorated calls for incentives to reduce GHG emissions, including the pricing of carbon and the levy of a carbon tax. However, the latest analyses on the subject [14,15] indicate that a tax on GHG emissions may lead to significant tradeoffs between emissions abatement and food security.

It is in this environment that the concept of Climate Smart Agriculture (CSA) has become increasingly relevant. CSA proposes a framework that supports decision-making in the agriculture sector by considering three foundational outcomes and by fully accounting for the trade-offs and synergies among them. It is comprised of agricultural systems that contribute to sustainable and equitable increases in agricultural productivity and incomes; greater adaptation and resilience to climate change of food systems from the farm- to the national-level; and reduction, or removal, of greenhouse gas emissions, where possible [16].

Many operational aspects of CSA are still under investigation as local contexts determine the enabling environment as well as the trade-offs and synergies between productivity, adaptation, and mitigation [17,18]. Farmers must identify what can be considered climate-smart given their biophysical, agricultural, and socio-economic context. As a consequence, the use of the CSA approach is knowledge-intensive and can require considerable institutional support [19,20]. Because of these difficulties, Chandra at al [21] note that, at this time, CSA is “a popular scholarly solution” experiencing difficulties in translating into smallholder farmer and civil society actions as well as new policy directions. Taylor [22] illustrates how the failure to incorporate issues related to social justice make the acceptance and implementation of CSA difficult in many communities. The frequent result is that, even when farmers, agrarian organizations, large scale farmers, and policy maker have embraced the concept of CSA, they struggle with the implementation and tend to look for simple protocols to follow.

Despite these unsettled issues, a substantial amount of resources have been mobilized to promote and implement CSA at a scale sufficient to have a global impact [23]. Even though CSA is more than a set of agricultural practices, it does include some specific technologies and agronomic tools, and these are the focus of this study. Our goal is to provide a first set of boundaries for the global effects of CSA used in food-crop production with a particular attention to its potential to reduce GHG emissions without jeopardizing food security. For this, we focus on some aspects of food-crop production that can be modelled globally with a reasonable level of accuracy given the latest developments in modeling capabilities [9,24–26].

Our analysis looks at four major categories of agricultural practices: no-till, integrated soil fertility management, nitrogen use efficiency and alternate wetting and dry. They have been shown to have positive impacts on yields and GHG mitigation across a wide range of conditions, but they all require specific modifications and adjustments on the ground. As a consequence, the modelling work presented here is a stylized representation of a range of many technologies and practices that would be identified using the CSA approach.

Notwithstanding these limitations, the results of our analysis clearly indicate that CSA practices have the potential to increase food production under unfavorable climate regimes and to improve the food security conditions of millions of people while reducing GHG emissions. However, results also indicate that for CSA to make a sufficiently large impact on global GHG emissions, it must be implemented widely across the entire sector and requires a significant amount of support and coordination. Our findings are also suggestive of broader benefits related to the resilience of the production system, to a reduced pressure for expanding cropland area and to reduced soil fertility depletion. While encouraging, these results are the product of the method and of the modeling assumptions used in the analysis and they must be evaluated and refined by additional global and regional analyses.

## 2 Methods

We performed an ex-ante analysis of the long-term effects on global food security and GHG emissions of adopting of a set of CSA technologies and practices to grow three widely grown crops: maize (*Zea mays*), wheat (*Triticum aestivum*), and rice (*Oryza sativa*). These three crops represent about 41% of the global harvested area and approximately 64% of the estimated 2–3 Gt CO_2_e per year emitted by crop production globally [27]. The effects of CSA practices on production, food security, and GHG emissions were assessed via comparison with the outcomes of a business-as-usual (BAU) scenario in which farmers retain the current practices during the period 2010–2050.

**Fig 1:**
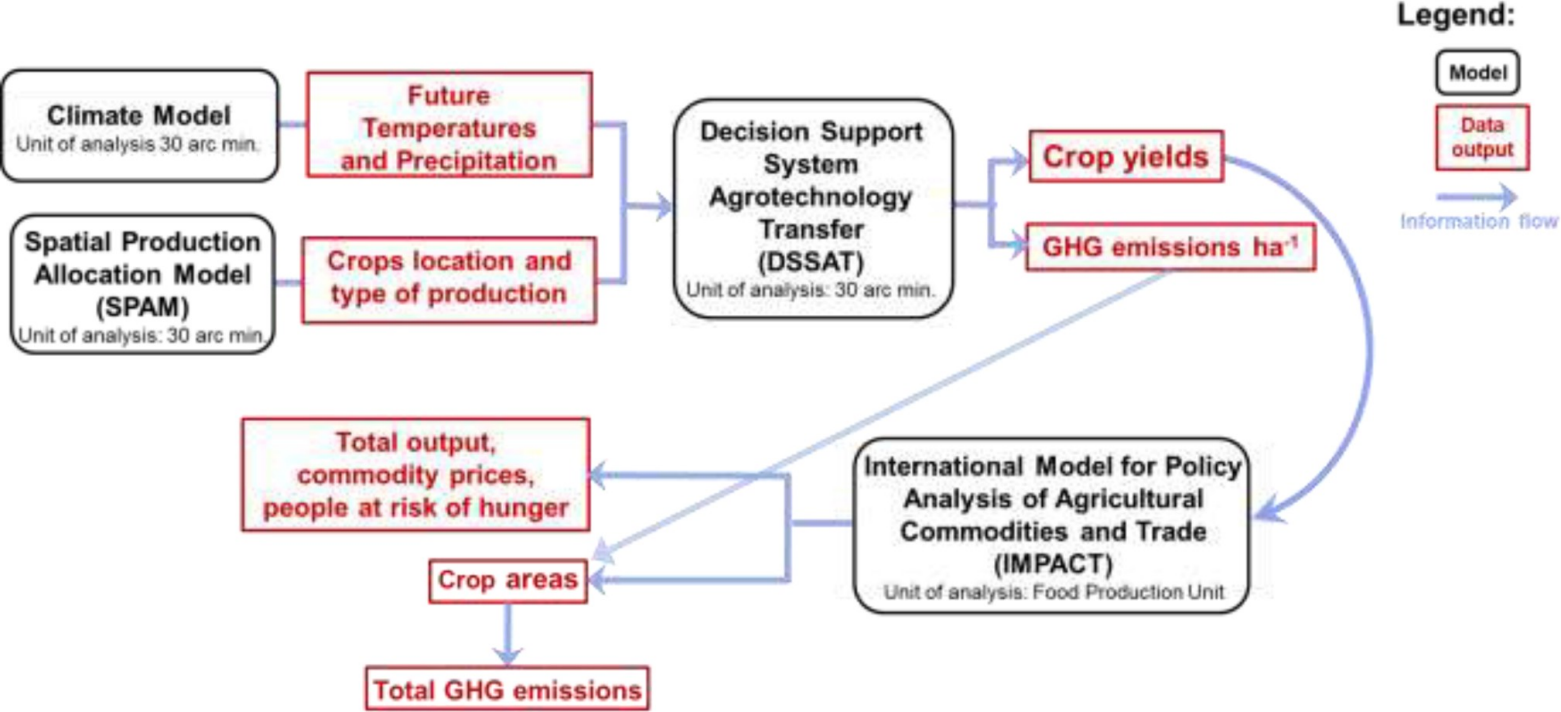
Scenario modeling steps and information flow. Source: Authors.

All scenarios, BAU and alternatives, were created using the IMPACT system of models [26,28] which links crop and climate models to the core economic model in which agricultural production is represented across 320 sub-national regions called “food production units” (FPUs, see Supplementary Discussion S1 in S1 File). Because of the linkages among these models, IMPACT’s outputs reflect the interactions between biophysical, economic and population trends, the combination of which represents the functioning of the global food market. A stylized representation of the modeling steps and of the information flow is provided in Fig 1. The process represented in the figure is used to generate each one of the scenarios described in the sections below.

### 2.1 Simulation of production, prices and food security—the core of the modeling system

To simulate the effects of CSA adoption on yields, production, harvested areas, world commodity prices and indicators of food security, we linked the spatially-disaggregated data of three models: the Spatial Production Allocation Model (SPAM) [29], the Decision Support System for Agrotechnology Transfer (DSSAT) [30], and the International Model for Policy Analysis of Agricultural Commodities and Trade (IMPACT v3.3) [28] (Supplementary Discussion S1 and Supplementary Fig 1 in S1 File).

The SPAM model was used to identify the location of crop production on a global grid in which each grid-cell is of size 30 arc minutes (a square of approximately 56 km by 56 km at the equator). For each grid-cell, SPAM provides a database cataloging the dominant crops, representative cultivars, management practices, and the inputs used. Data about climate, irrigation type, and soil properties were geo-linked to each SPAM grid-cell and were essential to run crop model simulations at the grid-cell level. The DSSAT crop model was used to simulate crop yields, with current and or with CSA practices, as a function of the interaction between biophysical elements of the crop systems (e.g. soil, weather, and crop) and management practices (e.g., tillage, nutrient application, and water availability). After the completion of a calibration step (Supplementary Discussion S2 with Supplementary Table 2 and Supplementary Figs 2 and 3 in S1 File), the yield responses were aggregated to evaluate agriculture production across IMPACT’s FPUs. IMPACT utilizes the yield responses to agricultural practices as shifters for the crop-specific supply curves and for the yield’s growth rates [1,9,28,31]. Starting from these changes in growth rates, the systems of equations at the core of the model endogenously determine crop areas, agricultural production, commodity prices and food availability. In turn, taking into consideration population and income growth, changes in food availability translate into changes in the regional availability of kilocalories [28]. In IMPACT, the share of people at risk of hunger (i.e. suffering from undernourishment) is calculated based on an empirical correlation between the share of undernourished people within the population and the relative availability of food. The calculation is adapted from Fischer et al. [28,32]. The share of undernourished children under the age of five is based on the calculation of the average calorie availability per capita per day, women’s access to secondary education, the ratio of female to male life expectancy at birth, and health and sanitation conditions [28,33]. It is an estimate of undernourishment in terms of weight for age.

### 2.2 Implementation of CSA technologies in DSSAT

The CSA scenarios simulate the use of four practices that are recurrently identified in the literature for their potential to deliver across the objectives of CSA and to be adopted widely [18,34]. There are several other practices and technologies that are proposed for CSA, the ones selected are among those that can be modeled with higher accuracy. The technologies considered for maize and wheat are no-till and integrated soil fertility management, while those for rice are alternate wetting and drying and nitrogen use efficiency. These practices are already utilized and widely tested due to their promising positive effects on yields, their higher resource use efficiency (e.g., water and nutrients) and their overall effects on GHG emissions (Table 1 and Supplementary Discussion S3 for a description of the technologies; Supplementary Discussion S4 and Supplementary Table S3 for modeling details and an assessment of our simulation results in S1 File).

**Table 1:** CSA technologies considered in this study.

CSA technology	Description	Crop	Potential effects on yields and GHG emissions	References
**No tillage**	Minimum or no soil disturbance, often in combination with residue retention, crop rotation, and use of cover crops	Maize, wheat	• Positive or neutral effects on yields • Uncertain effect on GHG emissions	[35–39]
**Integrated soil fertility management**	Combination of chemical fertilizers, crop residues, and manure/compost	Maize, wheat	• Positive effects on yields • Variable effects on GHG emissions	[40–43]
**Alternate wetting and drying**	Repeated interruptions of flooding during the season, causing the water to decline as the upper soil layer dries out before subsequent re-flooding	Rice	• Lower to no significant changes in yields. • High confidence in lower GHG emissions due to reduction of methane emissions	[44–48]
**Nitrogen use Efficiency**	Practices and technologies to enhance the uptake of inorganic soil nitrogen by crops	Rice	Ȣ Positive results on yields • Reduction of GHG emissions	[44,49,50]

Source: authors

In order to simulate no-till, the default setting for conventional tillage in DSSAT was removed and a seed planting stick and a deep fertilizer injection were used as planting and fertilizer application methods to minimize soil disturbance (more details in Supplementary Discussion 3 in S1 File). Six countries (Argentina, Australia, Brazil, New Zealand, Paraguay, and Uruguay) where no-till has already been widely adopted were excluded from the simulations [51,52] because we assume that the potential for expansion in these six countries is low. In other places, especially North America where no till is already present but not widely adopted, we assume that it is not utilized and therefore our analysis may overestimate the impacts of using this practice.

Integrated soil fertility management was implemented by simulating the application of organic amendment, in addition to the inorganic fertilizer applications already defined in the BAU scenario. The site-specific organic manure application quantity was based on Potter et al. (2010; http://www.earthstat.org) [53] and was used monthly during the fallow period (after harvesting–before planting). The rate of inorganic fertilizer application is the same as in the BAU scenario; however, the application scheduling is optimized based on the growth stage of each crop to minimize nitrogen stress during flowering and grain filling.

For rice production, enhanced nitrogen use efficiency and alternate wetting and drying (AWD) were simulated. Nitrogen use efficiency was simulated by focusing on enhanced plant uptake of nitrogen fertilizer under both rainfed and irrigated conditions. This was done by turning on in DSSAT the option for nitrogen fertilizer application based on deep placement of urea supergranules at a depth of 10 cm beneath the surface soil. Simulation of AWD was only applied to irrigated rice and was based on a recent study by the International Fund for Agricultural Development-IFPRI Partnership Program that identified alternative agricultural mitigation options for rice production using the DeNitrification-DeComposition model [DNDC; 54]. We employed the same modeling approach of DNDC, which assumes that: i) rice paddy is initially flooded to 10 cm, ii) water level is reduced at rate of -0.5 cm/day to -5cm, and iii) then re-flooded at rate of 0.5 cm/day till to 10 cm.

### 2.3 Calculation of GHG emissions

DSSAT was used to calculate per-hectare GHG emissions which were then multiplied by the harvested areas projected by the IMPACT model to compute total GHG emissions. Specifically, temporal changes in soil carbon stock were simulated in the CENTURY soil organic matter (SOM) module embedded into DSSAT [55]. Direct N_2_O emissions were simulated by modifying DSSAT’s source codes to model denitrification processes. The modifications ensure that our estimates of direct N_2_O emissions are comparable to those calculated using the 2006 Intergovernmental Panel on Climate Change (IPCC) emission factors [56], where 1% of N additions from mineral fertilizers, from organic amendments and from crop residues, 1% of N mineralized from soil organic matter, and ~0.7% of N from residue inputs are converted into N_2_O emissions.

For flooded rice soils, we used the IPCC default emission factor of 0.3% of applied N. Methane (CH_4_) emissions were calculated by combining DSSAT-simulated rice biomass with IPCC Tier 1 method’s emission coefficients proposed by Yan et al. 2009 [57]. Parameters of the Tier 1 method include baseline emission factor (1.3), scaling factors for continuous flooding (1) and multiple drainage (0.52), simulating effect of rice straw (0.59), and conversion factor of farmyard manure (0.14). These were combined with the simulated outputs of rice yields and straws, days in growing season, soil organic carbon content, and the input data of manure application rate. Finally, all GHG emissions were converted into tons of CO_2_ e using global warming potential for 100-yr time horizon of each GHG (Supplementary Table S4 in S1 File).

### 2.4 Scenarios

#### 2.4.1 Business as usual scenario

BAU scenario reflects the use of current practices and technologies throughout the 2010–2050 period and assumes that agriculture is developing under climate change conditions. BAU and all the alternative scenarios use the population and income growth assumptions that underly the middle of the road Shared Socioeconomic Pathway 2 (SSP2; [37,38]) from the IPCC Assessment Report 5 (AR5; [15,24,58]). Under the SSP2 narrative, global population will reach over 9 billion people by 2050 at an average annual growth rate of 0.6% and per capita GDP grows at just below 2% per year. The climate models used in both DSSAT and IMPACT under a Representative Concentration Pathway 8.5 (RCP 8.5; [59]) represent two possible future states of the climate. One, the GFDL-ESM2M (GFDL; [60]), is drier and cooler than the other, the HadGEM2-ES (HadGEM; [61]) (Supplementary Figs 4 and 5 and Supplementary Table S5 in S1 File). Given the high uncertainty around the overall effects of carbon fertilization on crop productivity [62,63] we cannot make conclusive statements about how increased concentrations of CO_2_ in the atmosphere will impact production (see a detailed treatment of the issue in Supplementary Discussion S5 in S1 File). Therefore, our modeling approach assumes no additional effects on yields from atmospheric carbon.

#### 2.4.2 Climate smart agriculture scenarios

These scenarios were constructed by assuming that farmers who are currently using a particular set of practices to grow maize, wheat, and rice are offered a portfolio of alternatives from which to choose (i.e. the four CSA practices considered). We first explored two scenarios with the objective to shed light on the largest possible effects of adopting CSA practices. The scenarios are based on two basic adoption rules implemented at the grid-cell level. The first (Rule 1) requires that CSA practices generate a yield gain compared to BAU in order to be adopted. The second (Rule 2) requires that an alternative practice generates higher yields and a decrease in emission intensity (i.e. the quantity of CO_2_ e per unit of product). If none of the alternatives increases yields, it is assumed that farmers retain their current practices. Given our objective, both scenarios assume that when a CSA practice is chosen all farmers in a given area adopt it beginning from the first year (i.e. 100% of the area in which Rule 1 or Rule 2 are satisfied it is assumed to adopt the CSA practices). Admittedly, the chosen adoption rules overestimate the role that yield gains play in farmers’ decisions. We do recognize that there are other benefits that might motivate adoption. However, at this time there are no widely applied financial mechanisms that disincentivize the use of current agricultural practices or that promote CSA [64]. Furthermore, non-monetary benefits vary greatly with local conditions and with farmers’ idiosyncrasies which cannot be represented in a global modeling exercise. More importantly, it is unlikely that countries would support the wide uptake of practices that reduce yields, given the importance that increased productivity holds for food security. We therefore think that, albeit with limitations, the assumption that adoption requires a yield increase is a pragmatic way to move forward in global analysis such as the one we are undertaking.

We did however test the possible effects of production costs and other barriers to adoption through two additional scenarios. It is well known that production costs and other factors (e.g. farmers’ access to markets and to credit, the characteristics of a particular technology, the quality of extension services, attitudes towards risk and risk exposure) affect the adoption of new practices and technologies [65–68]. Based on these notions, a third scenario was implemented which uses adoption Rule 1 together with the adoption rates used by Rosegrant et al. [9]. These implicitly include multiple costs related to adoption and were obtained through surveys and interviews with experts. These rates are more realistic than the 100% adoption assumed in the previous scenarios (Supplementary Discussion S6 with Supplementary Table S6 in S1 File).

The issue of costs of production is particularly important for AWD, a practice that could significantly reduce the use of water without reducing rice yields (see Table 1). This means that AWD could be adopted even though there is a reduction in yields. Therefore, the fourth and last simulated scenario uses Rule 2 but expands adoption by including the potential reduction of production costs associated with implementing AWD. A review of the literature reveals that irrigation costs represent from 3 to 36% of production costs [56,57,69] and AWD is reported to reduce irrigation cost up to 30% [70,58]. Based on these estimates we calculate that as long as yields decrease less than 9%, AWD is still be more profitable, and therefore preferable, to the current practices (Supplementary Discussion S7 in S1 File).

The performance of CSA practices depends on tailoring their implementation to the specific local conditions, but the capacity and knowledge to do so varies greatly from farmer to farmer. Therefore, for all scenarios we simulated instances in which farmers perform a poor, average, and optimal tailoring of the technology to their local biophysical circumstances (represented in the model by weather and soil characteristics). To do this, we exploited the fact that each IMPACT FPU contains multiple 30 arc minutes grid-cells. We considered the yield gain distribution in each FPU and used the average of the lower quartile, the average of the distribution, and the average of the upper quartile to represent a poor, average, and optimal tailoring of CSA.

Thus, our evaluation of the effects of widespread adoption of CSA practices is based on a total of 26 simulations (Table 2). Given the amount of output generated, for each scenario we report a single value which is the average of the GFDL and HadGEM results followed in parenthesis by the range of the results (lowest–highest value) obtained using the different climate models and levels of tailoring. The same information is indicated in the whisker bar in the figures.

**Table 2:** Summary of scenarios simulated.

Scenarios	Climate model	CSA tailoring	Socioeconomic & Emission assumptions	Simulation
BAU	GFDL	None	SSP2 + RCP8.5	1
	HadGEM	None		2
Adoption Rule 1: Higher yields than BAU	GFDL	Lower		3
		Average		4
		Optimal		5
	HadGEM	Lower		6
		Average		7
		Optimal		8
Adoption Rule 2: Lower emissions intensity & higher yields than BAU	GFDL	Lower		9
		Average		10
		Optimal		11
	HadGEM	Lower		12
		Average		13
		Optimal		14
Adoption Rule 1 and rates of technology uptake from Rosegrant et al 2014 [9]	GFDL	Lower		15
		Average		16
		Optimal		17
	HadGEM	Lower		18
		Average		19
		Optimal		20
Adoption Rule 2 plus AWD production costs	GFDL	Lower		21
		Average		22
		Optimal		23
	HadGEM	Lower		24
		Average		25
		Optimal		26

Source: Authors

## 3 Results

### 3.1 Prices and production

Projections for the BAU scenario indicate that global production of maize, wheat, and rice in 2050 will increase by 47% (36–58%), 42% (40–44%), and 19% (18–20%) respectively, compared to 2010. Prices are projected to increase by 80% (56–103%), 35% (24–46%), and 52% (44–60%) respectively. Therefore, despite the impact of climate change, production of these three main cereals is projected to increase. After economic growth and changing incomes and diets are considered, by 2050 according to BAU projections there will be 47 million (45–48) fewer undernourished children and 385 million (361–410) fewer people at risk of hunger.

**Fig 2:**
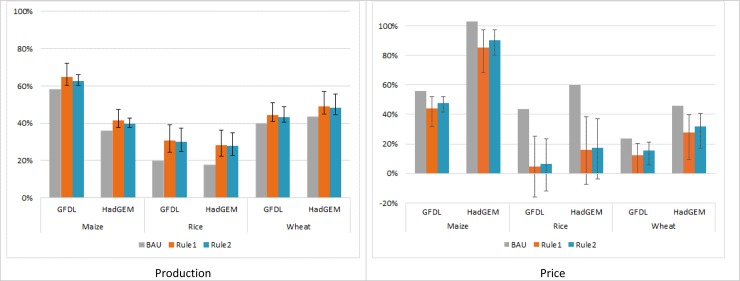
Percent change in production (total output) and prices comparing years 2010 and 2050 for BAU and CSA adoption scenarios. Columns indicate the average tailoring of CSA practices; whisker bars identify results for the poor and optimal tailoring. Source: Authors. BAU = business as usual scenario.

We first report in detail the results for the CSA adoption scenarios based on Rules 1 and 2. The sensitivity of these scenarios to barriers to adoption and to costs of production is reported later in the paper. The results show that CSA practices are adopted on a total of approximately 372 million hectares when adoption is based exclusively on yield increase (Rule 1) and on 241 million hectares when adoption is dependent on reduction in emission intensity and increase in yields (Rule 2) (Supplementary Discussion S8 with Supplementary Table S7 in S1 File). Compared to BAU, by 2050 CSA practices are estimated to increase global production of maize by an additional 4% (1–9%) with Rule 1 and 3% (1–5%) with Rule 2; wheat production is also estimated to increase by about 4% (1–9%) with Rule 1, and 3% (0.4–8%) with Rule 2. CSA practices appear to have the largest effect on rice, for which production is projected to increase by about 9% with both rules (4–16% with Rule 1 and 4–15% with Rule 2) (Fig 2).

We should note that not all countries experience an increase in production. The wide-scale adoption of CSA practices induces a reorganization of global production because of differences in land suitability. As a result, in some countries the reduction in crop harvested area offsets the gains in yields (Supplementary Table S8 in S1 File). Nevertheless, overall the changes in production are sufficient to have a sizable impact on world prices (see description of endogenous effects in Supplementary Discussion S9 in S1 File). Prices are still projected to increase but compared to BAU their growth is reduced by 8% (3–17%) for maize, by 11% (3–25%) for wheat, and by 27% (13–42%) for rice with Rule 1, and by 6% (2–11%) for maize, 8% (2–20%) for wheat and 26% (14–40%) for rice with Rule 2 (Supplementary Table S9 in S1 File). As a result, the population at risk of hunger decreases more than what is projected by the BAU scenario. The number of people at risk of hunger is reduced by an additional 34 million (10–69 million) by 2050 under Rule 1 and by 29 million (10–59 million) under Rule 2, with the largest improvements in Sub-Saharan Africa, East Asia and Pacific, and South Asia. Similarly, the number of undernourished children decreases by an additional 2 million under both Rules 1 and 2 (a range of 1–5 million with Rule 1 and 1–4 million with Rule 2) with most of the improvements in Sub-Saharan Africa and South Asia (Supplementary Table S10 with Supplementary Figs 6 and 7 in S1 File).

### 3.2 GHG emissions

Global GHG emissions decrease under both adoption scenarios, but there are important distinctions between the two. When farmers’ adoption choices are based only on yield increases (Rule 1), the reduction in GHG emissions is estimated to be equivalent to 44 Mt CO_2_ e yr^-1^ (9–77 Mt CO_2_ e yr^-1^). The reduction of emissions is significantly higher with Rule 2, 101 Mt CO_2_ e yr^-1^ (84–124 Mt CO_2_ e yr^-1^). This shows that there is a substantial amount of area in which CSA practices can increase yields but do not reduce GHG emissions. The higher levels of emissions abatement come at the cost of production. On average across climate scenarios, under Rule 2 total cumulative production for the three crops is reduced by 21 Mt yr^-1^ (1 and 53) of fresh matter harvest compared to Rule 1. This is equivalent to 1% (0.1 and 2%) of total yearly global production of maize, wheat and rice.

**Fig 3:**
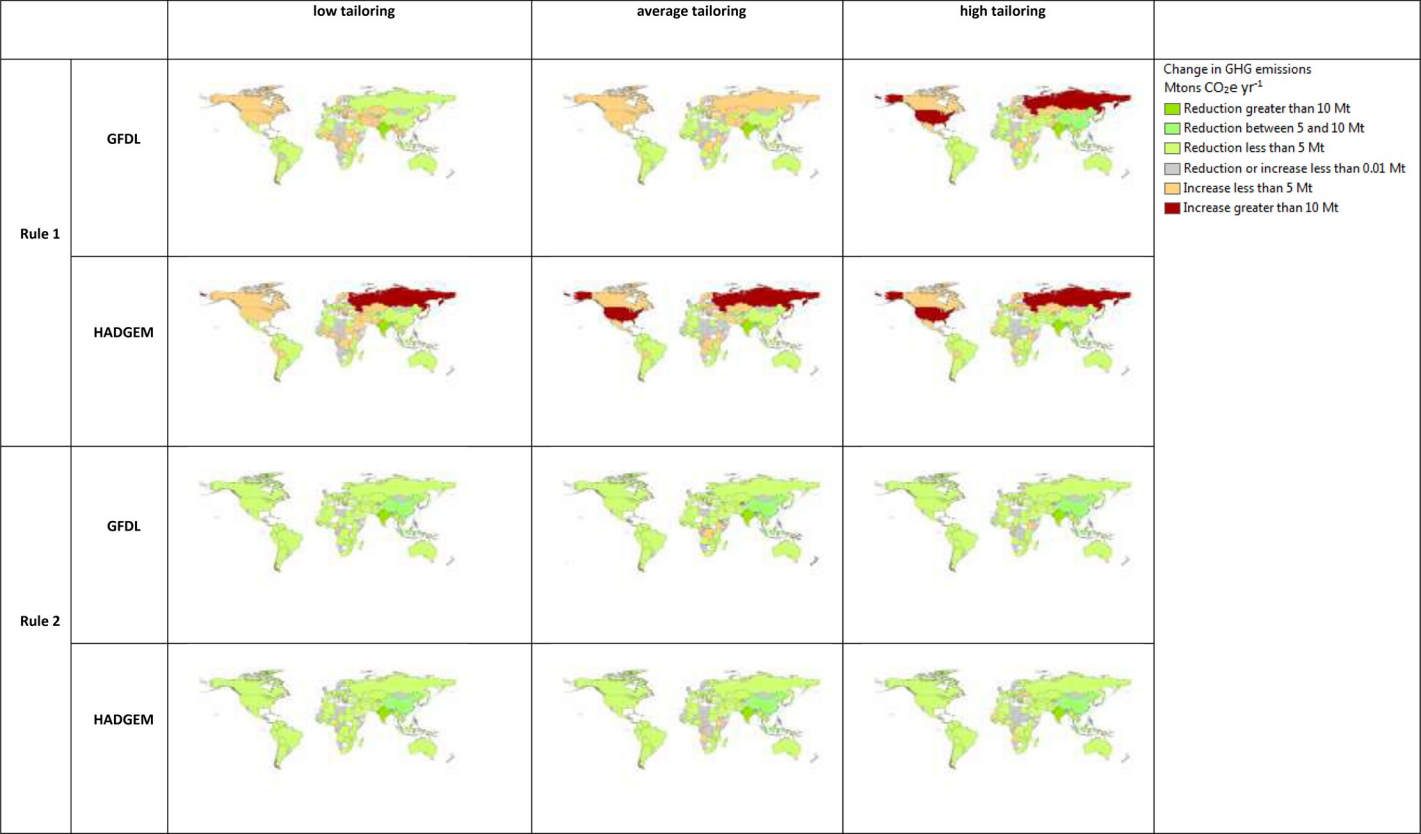
Average change in GHG emissions by country for alternative scenarios and tailoring of CSA practices, comparing years 2010 and 2050. Negative values indicate an abatement compared to BAU and positive values an increase. Source: Authors.

It is important to note that the effects on emissions vary from country to country and that we find instances in which an increase in emissions occurs in some countries (Fig 3 and Supplementary Table S11 in S1 File).

Emissions can increase because of the potentially large changes in countries’ crop harvested areas and yields. Total emissions can increase even when a reduction of emission intensity is achieved if the reduction in emissions per unit of output is offset by increases in yields or in areas (Supplementary Discussion S10 with Supplementary Table S11 in S1 File).

### 3.3 Other effects related to emissions

Interestingly, part of the reduction in emissions is due to the effects on soil organic carbon concentration, which is estimated to grow compared to BAU by 0.11 t ha^-1^ yr^-1^ (0–0.49 t ha^-1^ yr^-1^) over the area that adopts the alternative practices based on Rule 1, and by 0.14 t ha^-1^ yr^-1^ (0.01–0.53 t ha^-1^ yr^-1^) based on Rule 2. These changes are beneficial for sustainable production and resilience since higher soil organic carbon concentrations increase soils nutrient availability and soils water retention.

Some potentially important effects on land use must be noted. The combination of higher yields and lower prices reduces producers’ incentives to expand production onto additional land as the demand for wheat and rice can be satisfied with less harvested area (Supplementary Table S12 in S1 File). Even though global harvested area for maize is projected to expand by 1 million hectares in 2050, on average across Rule 1 and 2 the net effect is a decrease in total harvested areas for the three crops estimated at 10 million hectares (3–27 million hectares) compared to BAU. This result is suggestive of a reduced pressure on forests and other natural areas that might be environmentally significant and rich in carbon. However, the reallocation of harvested area following changes in production and prices causes other crops to take over the land freed by rice and wheat (Supplementary Discussion S11 in S1 File). Total land allocated to soybeans, vegetables, temperate fruits, sugarcane, and rapeseed is estimated to increase by 2 million hectares in 2050 on average across Rule 1 and 2 (1–4 million hectares) (Supplementary Table 13 in S1 File). Depending on how they are grown, these crops might have a higher carbon footprint than the crops they replace.

Importantly, simulations show that increases in production of grains reduces the price of livestock feed and increases the number of animals that can be supported globally. The stronger the effect on prices, the greater the increase in the global cattle herd. As a result, emissions from cattle may increase by 5.4 Mt CO_2_e yr^-1^ (1.2–13.1 Mt CO_2_e yr^-1^) by 2050 and partially offset the reductions in emissions discussed earlier (Supplementary Discussion S12 and Supplementary Table S14 in S1 File).

### 3.4 Adaptation and resilience

The effects on production, prices, soil, and land use suggest that CSA practices are a form of adaptation to new climate conditions and make crop-production more resilient. A comparison with a scenario in which the effects of climate change on future crop production are removed (a No Climate Change scenario. See Supplementary Discussion S13 and Supplementary Fig 8 in S1 File) show that the production gains obtained from CSA practices can offset the negative impacts of climate change on maize and rice production and slow down consequent increases in prices. CSA practices are also successful in reducing the price of wheat which, despite an increase in production, is projected to increase in the BAU scenario. It is however difficult to draw broader conclusions about resilience, as this would require a more specific analysis of the differential effects across multiple social contexts, at different geographical scales, and for different social groups [71,72].

### 3.5 Effects of adoption rates and production costs

**Fig 4:**
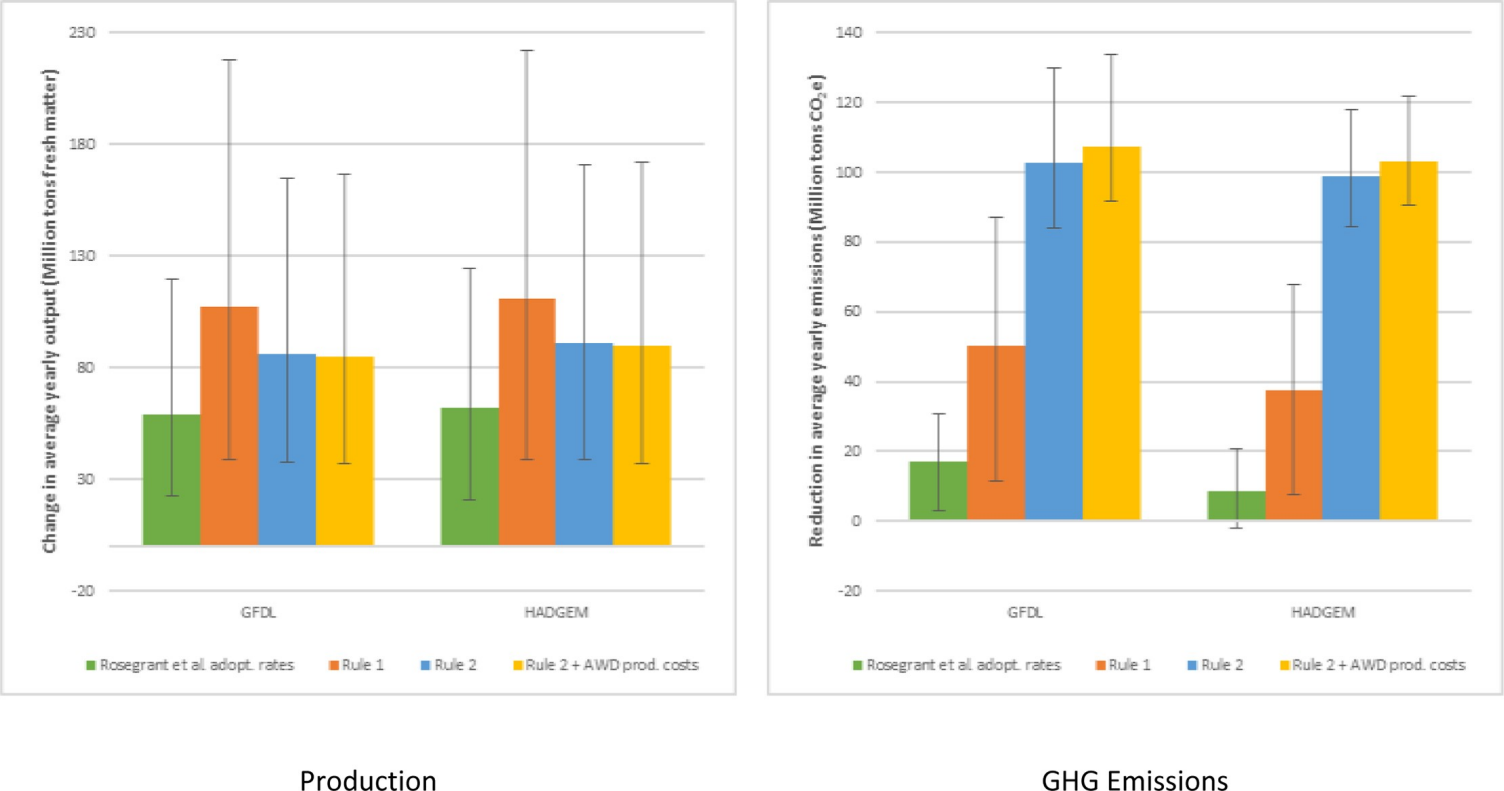
Changes in average yearly cumulative production of maize, rice and wheat and GHG emissions for alternative scenarios for the period 2010–2050. Columns indicate the average tailoring of CSA practices; whisker bars identify results for the poor and optimal tailoring Source: Authors.

Results for the third scenario, which uses lower adoption rates, and the fourth, which includes AWD production costs, are reported in Fig 4 along with the results from Rules 1 and 2. To provide a comprehensive review of all the simulated scenarios, we consider the cumulative fresh weight harvest for the three crops. The results in Fig 4 focus on production and emissions. Similar results for harvested area are presented in Supplementary Table S15 in S1 File.

As expected, social and cost barriers—heuristically included in the simulations by reducing the rate of adoption of CSA practices as in Rosegrant et al [9]—significantly affect the results with an overall reduction in benefits. Compared to BAU, yearly production for the three crops increases on average by 60 Mt (22–122 M tons) and GHG yearly emissions are reduced on average by 13 Mt CO_2_e (-2–31 Mt CO_2_e, where the negative number indicates an increase in emissions). Therefore, the reduced adoption lowers the crop production gains observed using Rule 1 by about 40% and reduces the effects on emissions by two thirds or more.

When the reduction in production costs for AWD is accounted for, AWD is adopted on some additional 0.8 million hectares leading to the highest achieved emission reduction. Yearly production is essentially unaffected but yearly abatement reaches on average 105 Mt CO_2_ e yr^-1^ (90–134 Mt CO_2_e yr^-1^).

## 4 Discussion

The largest positive impacts on production and food security as well as the highest levels in GHG emissions abatement, should be interpreted as aspirational targets and viewed as an upper bound of the possible effects of adopting the CSA practices considered across maize, wheat and rice. These results are predicated on high levels of uptake by farmers and their capacity to tailor the implementation of CSA practices to local conditions. The scenario that simulates lower and more realistic adoption rates and the scenarios that represent a lower proficiency at using the practices, show rapidly diminishing benefits. This stresses the importance of major new investments to overcome long-standing problems such as underperforming extension services, farmers’ lack of credit, risk management and timely information about markets. These barriers are known to prevent the adoption of more productive, more resilient and sustainable agricultural systems.

Even though the focus of CSA is not on mitigation benefits, the pressure on the agriculture sector to reduce GHG emissions is likely to increase as other sectors reduce their share of global emissions. The performance of agricultural practices in terms of their abatement potential is therefore important. According to our assessment, the total maximum abatement obtainable (134 Mt CO_2_e yr^-1^) is about 17% of what is considered the economically achievable mitigation from managing cropland, which is 0.77 Gt CO_2_e yr^-1^ [73]. Also, after the indirect effects of cattle emissions are accounted for (approximately 4 Mt CO_2_e yr^-1^), the maximum abatement obtainable is 13% of the 1 Gt CO_2_e yr^-1^ abatement goal for the agriculture sector to remain below the 2°C global warming [74]. If one considers that the GHG emissions from the three crops analyzed is in the range of 1.28–1.92 CO_2_e yr^-1^ [25], our results point to a 7–10% reduction at best of those emissions. Given these limited abatement levels, the large scale adoption of alternative production systems (e.g. silvopastoral systems, agroforestry practices, precision agriculture) should be considered, and additional opportunities for abatement ought to be found elsewhere along the value chains [75].

Importantly, results indicate that recent concerns expressed in the literature regarding the negative effects of carbon tax on food security might be misplaced. In actuality, carbon pricing could help internalizing the external costs of GHG emission and steer the agriculture sector towards more carbon-efficient methods of production and distribution. Clearly, one can always impose a sufficiently high carbon tax with detrimental effects on the food security of a significant share of the population. However, our results for population at risk of hunger show that alternative practices and technologies, of which only a sample is explored in this study, can limit these effects if not completely offset them. In our analysis we did not explicitly explore the effects of policies that promote a reduction of GHG emissions. However, the results obtained using adoption Rule 2 show that as the emphasis shifts from yield gains to reducing emission intensity, emission abatement increases albeit at the cost of agricultural output. Resolving the tradeoff between emissions reduction and production in an economically efficient manner depends not only on carbon pricing or emission-reduction incentives but also on a proper pricing of the factors of production. An appropriate pricing of inputs like water and nitrogen fertilizers would promote the adoption of water-saving practices such as AWD with no significant reduction of productivity, and the adoption of practices that increase nitrogen use efficiency, with a consequent reduction in emissions and an increase in productivity.

Results also show the importance of understanding how changes caused by the widespread adoption of CSA practices may play on a global scale. The indirect effects arising from the reorganization of global production can lead to larger agricultural areas being allocated to other crops, and to an expansion in the global livestock herd due to cheaper feedstock prices. These effects can be large enough to limit the emission abatement effectiveness of CSA practices. In addition, the heterogeneous changes in countries’ GHG emissions, even in the presence of a global positive outcome, shows the importance of global coordination. Such coordination should also consider the interaction between agricultural land and carbon-rich environments such as forests and peatlands to avoid emission leakage [76,77].

Beyond the specifics of CSA, our results are strongly suggestive that using alternative practices the agriculture sector can increase its output and reduce its carbon footprint under future climate scenarios. However, many are the assumptions that underly our work and much more research must be undertaken to evaluate the global effects of changes in food production systems. Simulating alternative agricultural futures requires a model representation of the main structural drivers of demand and supply of food products and this requires significant assumptions about producers’ and consumers’ behavior. Scenario analyses based on model simulations, of which this study is an example, are not a prediction of the future. They are a representation of possible alternative futures given our current knowledge and assumptions about trends in climate, technology, population, income and other drivers, and about how they may interact in an economic system. Scenarios also rely heavily on existing global datasets, which come with many limitations. One way to test the robustness of the scenarios produced by models that do not generate confidence intervals or goodness-of-fit metrics, like the one we used, is to undertake a sensitivity analysis for some of the key variables. We have done this for climate, adoption levels, and yield performance but many more could be explored. Results from the sensitivity analysis point to the qualitative robustness of some of the findings (e.g. agriculture can withstand the negative impact of climate change using alternative practices, sustainable increase in production while reducing the carbon food print is possible), others indicate where additional research is necessary (e.g. investigate the multiple pathways that link agricultural output with the food security status of vulnerable people, move beyond the emphasis on yield gains and model the effects of crop rotations). Other results reveal the limits of the modeling environment that was used. A perfect example of this are our findings about land use change. The overall effects of higher yields and lower prices is to reduce the need for additional harvested area to fulfill the increasing demands for maize, wheat and rice. This suggests a reduced pressure to expand cropland and potential land-sparing effects. However, IMPACT does not model explicitly the competing demands of all land uses. It only considers cropland area and its agricultural output. While the idea that agricultural intensification reduces agricultural land’s encroachment into other natural areas is not new, it was famously put forward by Borlaug [78], whether this happens in reality is the subject of some controversy [79–81] and our model cannot directly resolve these questions. More research in this issue is therefore necessary.

One way to verify and test the validity of these scenarios is also to use alternative models after proper calibration [82] and compare results across them. Although this requires considerable resources, these types of comparisons have been done to assess global issues related to climate change such as agricultural production under future climate regimes [82], agricultural production and mitigation [83], and future nutritional challenges [84]. It would be advisable that a similar exercise is undertaken to explore the merits and benefits of alternative production systems like the one proposed by CSA.

## 5 Conclusion

Much has been written about CSA and its potential benefits but most of the existing analyses are based on local experiences and no study has so far attempted to quantify these benefits on a global scale. Our results show that widespread adoption of CSA practices can increase production and lower world prices of wheat, maize, and rice under future unfavorable climatic conditions. The reduction in prices is projected to make food products more accessible to millions of people thereby lowering the number of people at risk of hunger and that of undernourished children. These gains can be obtained while improving soil fertility and with a reduction in GHG emissions. Taken all together, results suggest that CSA practice can deliver benefits across its three foundational pillars on a planetary scale.

However, what clearly transpires from our results is that to make a significant impact, the principles of CSA must be applied widely across production systems and for this to occur significant investments must be made. Ideally, as others have suggested [85], the same or similar principles should be applied across the whole food system (i.e. trade, stocks, nutrition and social policies). It is also clear that the wide-ranging and multidimensional effects, sometime unintended or unforeseen, must be understood and managed. CSA with its multi-objective approach may provide a useful framework for decision-making ranging from the farm to the policy level.

## Supporting information

S1 File (DOCX)
