# Supplementary material for: Climate smart agriculture and global food-crop production
Source: PLoS One. 2020 Apr 29;15(4):e0231764. doi: 10.1371/journal.pone.0231764 (PMC7190105; doi:10.1371/journal.pone.0231764)
Supplement: S1 File — (DOCX) [file pone.0231764.s001.docx]

Supporting Materials

**Title**

Climate Smart Agriculture and Global Food-Crop Production

**Supple****mentary discussion**

S1. The IMPACT system of models

The IMPACT system of models links together the output of climate models, process-based crop simulation models, global hydrology models, and the IMPACT global economic model (Figure S 1). Crop models use information on geographical distribution of the crops under study (maize, rice and wheat) as well as their water management (rainfed or irrigated) from the Spatial Production Allocation Model (SPAM).

The DSSAT model

This study uses the Decision Support System for Agrotechnology Transfer (DSSAT) process-based crop models ^1^ to simulate the yield response of crops to baseline technologies and to the adoption of the climate-smart agriculture (CSA) technologies. Process-based models like DSSAT rely on decades of accumulated research on agronomy, soil science, and crop physiology and allow to represent explicitly all the constituent processes (soil, water, plant, management practices and so forth) and facilitate the simulation of their interactions. DSSAT has been used for decades by researchers, extension workers, and decision-makers to improve agricultural practices at the farm level, and to assess regional effects of climatic variability ^2–6^ and it is available at (<https://dssat.net/about>). The list of input and output data for DSSAT are described in Supplementary Table S 1.

The SPAM model

The Spatial Production Allocation Model (SPAM) uses crop suitability assessments, information regarding population density, and any other available prior knowledge regarding the geographical distribution of specific crops or crop systems to spatially allocate sub-national statistics of crop production and cropland data (period 2004-2006) at two levels of geographical disaggregation: 0.083 and 0.5-degree grid-cells (respectively 5 arcminute and 30 arcminute)^7^. For each grid-cell identified as a production area, the model provides a database of the existing (baseline) dominant management practices and inputs for maize, rice, and wheat (varieties, inorganic fertilizer application rate, organic amendment availability, and water management) and data on irrigation and soil properties. All the input data for the SPAM model is detailed in Table 2-1 of the SPAM technical documentation^8^.

SPAM is downloadable from the IFPRI Dataverse at <https://dataverse.harvard.edu/dataset.xhtml?persistentId=doi:10.7910/DVN/DHXBJX> and the dedicated SPAM website at <http://mapspam.info/> .

The IMPACT model

At the core of the system of models there is “International Model for Policy Analysis of Agricultural Commodities and Trade” model (IMPACT). IMPACT is a partial-equilibrium economic model that simulates national and global markets of agricultural production, demand, and trade associated with 62 agricultural commodities across 159 countries.

IMPACT combines projections data for both population and income, with yields data simulated through crop models, estimates of water availability from water models, and estimates of changes in temperature and precipitation from climate model. Therefore, its outputs reflect changes that come from the interaction of both biophysical and economic factors.

Simulated changes in yields through the DSSAT crop modeling suite provide to IMPACT an estimate of the effects of temperature-changes on productivity; effects on water availability are captured through linked water models ^9^. The IMPACT model gives and receives feedback from three water models (global hydrologic model, water basin management model, and water allocation and stress model), which can reflect the impact of climate change or policy decisions on the hydrology, or water allocation, thereby allowing to simulate changes in water availability for irrigation and their effects on agricultural production. Agricultural production is specified by models of land supply, and by allocation of land (irrigated and rainfed) to crops. Production is modelled at sub-national level, across 320 regions called “food production units” or FPUs. Additionally, it receives information on yield responses from crop simulation models (e.g. DSSAT).

The main drivers of the baseline suite of IMPACT scenarios (i.e. BAU scenarios) are GDP, population, and intrinsic agricultural productivity growth (IPR). GDP growth is obtained from the OECD ^10^ and population growth from IIASA ^11^. The choices of GDP and population growth are made to allow the IMPACT model to reproduce the Shared Socioeconomic Pathways (SSP scenarios) adopted by the Intergovernmental Panel on Climate Change Fifth Assessment Report (IPCC AR5). The intrinsic yield growth rates (IPRs) are based on past trends and expert opinion. Details of all the input data to the IMPACT model, and relative citations, are displayed in Table C1 (Appendix C) of the IMPACT documentation^12^

For extensive details on the equations at the core of the model, and information on data inputs especially population, GDP and yield growth (IPRs), refer to the IMPACT model documentation^12^. IMPACT has a long record of applications and it has been employed in a wide range of analyses, from assessing the potential effects of climate change on global food production and nutrition ^5,13^, to explore linkages between agriculture production and food security at the national and regional levels ^14–17^, to interdisciplinary assessment of economic models ^18,19^ to evaluating the global effects of biofuels production ^20^, to the assessment of economic effects of alternative mitigation policies ^21^ and the global simulation of technology adoption ^6^.

IMPACT is a partial equilibrium model and therefore it does not capture the economy-wide effects of income changes and consequent changes in consumers’ purchasing power. However, a quick review of the changes in countries GDP caused by adoption of CSA practices reveals that changes are still a small fraction of the entire GDP and overall effects on incomes outside the agricultural sector are small.

S2. Calibration of DSSAT

DSSAT’s simulation performance of three major crops was calibrated by adjusting model parameter and inputs so that DSSAT yields can be comparable with the grid-level SPAM yields, which are derived from FAO’s country-level statistics in 2005.

First, one model parameter (SLPF, a growth reduction factor on a scale of 0 to 1) and two model inputs (planting density and N fertilization rate) were used for DSSAT calibration. The former was chosen to account for the effects of the deficiencies of P and K and micro-nutrients on daily plant growth rate that DSSAT is not capable of modeling yet.

Second, a value of each parameter or input was changed using three levels. For example, the SLPF was assigned a value of either 0.6, 0.8 or 1.0, whereas planting density and N rate were assigned either the original values derived from the DSSAT input database or 50% or 150% of these original values. These levels resulted in total 27 possible combinations of model parameter and input values for each grid cell.

Third, SPAM yields were considered as “field-observed” yields for the calibration of DSSAT. These yields are represent harvested production and are expressed as fresh matter weight. They were therefore converted into dry-matter weight to be used in DSSAT. For this conversion, we accounted for harvesting and threshing losses defined as production minus harvesting and threshing losses per unit of harvested area and corrected for grain moisture contents (15.5, 13.5, and 13% as grain moisture contents for maize, wheat, and rice, respectively).

Finally, DSSAT was ran to simulate yields corresponding to all these combinations for the five continuous years of 2001 to 2005, followed by selecting the best combination of parameter and input levels that gave the lowest relative difference (RD) between simulated and observed yields (Yield_sim_ and Yield_obs_):

$\mathrm{RD}= \frac{\bar{Yield}_{sim}^{i}-\bar{Yield}_{obs}^{i}}{\frac{\bar{Yield}_{sim}^{i}+\bar{Yield}_{obs}^{i}}{2}}$ Eq 1.

Where $RD$ indicates relative difference, $i$ is an identification number of each 0.5-degree grid cell (30 arcminute), and $\bar{Yield}^{i}$ is a five year-average of yield for a grid cell $i$.

However, the value of RD at each grid cell can be a large number (either positive or negative) even with alternative combination of parameter values in DSSAT, especially when DSSAT simulates very low crop yields. To avoid this problem, RD were calculated only from grid cells where simulated yields are higher than minimum countries-wide yields reported in SPAM (Supplementary Table S 2). Then, a statistical test ^22^ was conducted to identify outlier grid cells (both irrigated and rainfed, for each crop). This test, based on RD, identifies those cells for which DSSAT is not able to simulate yields comparable to the “observed” SPAM yields. These outliers were eliminated from analysis.

After the calibration process, 42%, 45% and 47% of the total number of rice, wheat and maize pixels are retained, respectively. Regional representation is affected differently by this process. For maize cropland, about 80% of the original pixels belonging to the North America region are maintained after filtering, but only 20% are kept across sub-Saharan Africa. For rice, East Asia pacific is one of the regions with the largest number of retained pixels, with 54% of the total, whereas the number drops to 24% for Europe and central Asia (ECA). For wheat, 46% of the pixels survive the filtering process across South Asia and 30% in the Eastern Europe and central Asia region.

When the simulated yields after calibration were aggregated into country boundaries and compared to FAO’s country-level yields, simulated yields for maize and wheat are comparable to FAO yields with very good fits (R^2^ = 0.87 and 0.75 respectively); the fit for rice is lower (R^2^ = 0.63) is still acceptable (Figure S 2).

It must be noted that only monoculture systems were simulated. We acknowledge that this is a stylized representation of reality, which should be addressed in future research through inclusion of intercropping and rotation schemes.

Out of the total 320 food production units used in IMPACT, the loss of pixels results in the loss of maize data for 17 FPUs, rice data for 16 FPUs and wheat data for 14 FPUs (Figure S 3). By comparing the harvested area and production data from these FPUs to the world totals for harvested area and production in 2015, we see that the lost FPUs for maize accounts for 0.7% of global harvested area and 0.5% of global production. For rice, the dropped FPUs account for 0.6% of global harvested area and 1.3% of global production, and for wheat, the dropped FPUs account for 1.6 % of global harvested area and 2.3% of global production.

S3. CSA technologies and their implementation in DSSAT

Four practices and technologies are implemented in DSSAT and used in the simulations across all models. The technologies applied to maize and wheat are no-till and integrated soil fertility management (ISFM). For rice we implemented alternate wetting and drying (AWD) and nitrogen use efficiency (NUE). To provide some context, we first briefly report what the literature says about the on-farm application of these practices and technologies, and then we describe how they were implemented in DSSAT.

Unlike continuous tillage, which leaves soils prone to soil erosion and is a major source of soil carbon loss ^23^, no-till combined with crop rotation and retention of crop residues reduces erosion and improves general soil fertility through retention of water and nutrients, and benefits to soil aeration and soil biota with potential direct effects on agriculture productivity ^24,25^. Integrated soil fertility management (ISFM) uses crop residues along with both synthetic fertilizers and organic inputs (e.g., animal manure and/or green manure), aiming at increasing productivity through the efficient use of nutrients ^26^. AWD has been used in paddy rice cultivation, which after livestock and soil is one of the main sources of non-CO2 greenhouse gas (GHG) emissions from the agriculture sector ^27^, to significantly reduce CH_4_ emissions from rice paddies ^28,29^. NUE can be broadly defined as a practice or technology to enhance the crop uptake of soil nitrogen. In this study, we define NUE agronomically, focusing on technology to increase the recovery of nitrogen fertilizer. Specifically, we use the deep placement of urea fertilizer as a representative example for NUE. Urea deep placement (UDP) is a technique in which urea briquettes are placed directly in the plant root zone to improve the efficiency of nitrogen use, which is the key to increase production, minimize environmental impacts, and reduce emissions ^28^. Broadcast application of nitrogen in rice fields leads to 60 to 70 percent nitrogen losses, which directly contributes to both water pollution and GHG emissions. The placement of urea briquettes, or ‘supergranules’, deep in the soil provides a slow release of fertilizer near the root system of planted rice, therefore improving the efficiency of nutrient uptake and limiting nitrogen losses.

For wheat and maize production, no-till and integrated soil fertility management (ISFM) were implemented as CSA technologies, both under rainfed and irrigated conditions. Simulation of no-till was set up by removing the default-conventional tillage practice in DSSAT. To minimize soil disturbance, a seed planting stick was simulated as the planting method. No-till practice requires farmers to minimize soil disturbance especially during the phase of seed-planting. In the DSSAT crop model, tilling the soil is the default seed-planting practice, and as a result the model simulates crop growth under conditions of soil disturbance. For example, when the DSSAT input file instructs the model to plant seeds at 5 cm depth, DSSAT automatically simulates soil disturbance to a 5 cm depth, with loss of soil organic carbon as a consequence. To prevent this effect, and implement no-till in our study, we specifically instructed DSSAT not to disturb soil even during the planting, as if the seeds were precisely planted by a thin stick. For fertilizer application, a deep injection method parameter available in DSSAT was simulated under no-till. Six countries (Argentina, Australia, Brazil, New Zealand, Paraguay, and Uruguay) where no-till has been widely adopted were excluded from simulation of no-till. ISFM was implemented by applying organic amendment in addition to the inorganic fertilizer applications defined in the baseline management scenario.

NUE and AWD were simulated as CSA practices applied to rice production. For NUE, which focuses on the enhanced plant uptake of nitrogen fertilizer, we used urea deep placement (UDP) as a representative technology, under both rainfed and irrigated conditions. UDP was simulated by selecting the in-built option for N fertilizer application method in DSSAT: deep placement of urea supergranules at a depth of 10 centimeters beneath the surface soil. Simulation of AWD was only applied to irrigated rice and was based on a recent study by the International Fund for Agricultural Development-IFPRI Partnership Program.

S4. Evaluation of modeled yields and GHG emissions

The reliability of model simulations for both productivity and emissions was tested by comparing our results to data from worldwide field experiments, through an extensive literature review. We looked at changes in yields reported from adoption of the technologies used in this study as well as absolute values and changes in GHG emissions under BAU and/or relative changes of GHG emissions under CSA practices. Table S 3 shows that the magnitude and direction of simulated results is comparable to results in the literature.

# S5. The issue of CO_2_ Fertilization

The extent to which elevated CO_2_ concentrations may benefit crop yields and offset the negative impacts of mean temperature and precipitation changes is still debated ^30–33^. The general expectation is that CO_2_ may provide a larger photosynthetic benefit to C3 crops (e.g., wheat, rice, soy) than to C4 crops (e.g., maize, millet, sorghum, sugar cane, but both C3 and C4 appear to achieve higher water-use efficiency at higher CO_2_ concentrations ^34^. A recent multi-ensemble crop model analysis found that higher CO_2_ concentrations (specifically under RCP8.5) may lead to an increase in wheat yields and protein content (although the results varies by region), but most of the actual gains may be negated by the increase in temperature and changes in precipitation ^33^. A 2014 review of available FACE (Free air CO_2_ enrichment) experiments also found “equivocal increases in net primary productivity (NPP) from [elevated] CO_2_ studies” ^35^. Overall, there is still significant uncertainty in the response of crops to CO_2_ especially in the long term ^36^ . The uncertainty stems mainly from a lack of field experiments and observations ^34,37^. As a consequence, crop models treat the CO_2_ effects in very different manners and the results can be seen in the high level of uncertainty in crop responses to CO_2_ fertilization across the full suite of crop models, included in the Global Gridded Crop Model Intercomparison (GGCMI) project (part of the Agricultural Model Intercomparison and Improvement Project (AgMIP)) ^38^.

Most crop models include relationships that simulate sensitivities of photosynthesis, dry matter growth, water use, reproductive process, and grain yield to the rising atmospheric CO_2_ concentration and a range of temperatures, which allow for the simulation of climate change impacts and potential farmer adaptation to climate change in both rainfed and irrigated agricultural systems. For the crop models in DSSAT specifically, Boote et al. (2011)^39^ provides a comprehensive review of literatures that evaluated the models’ response to CO_2_ fertilization, including the leaf-level photosynthesis rate changes (i.e., the efficiency of fixing CO_2_), taking into account the night-time temperature, leaf nitrogen concentration, and specific leaf mass changes, as well as the elevated CO_2_ concentration. Various studies found that DSSAT crop models adequately predict CO_2_ response to single-leaf and canopy assimilations. For the end-of-season grain yields, Boote et al. (2011) conducted a meta-analysis based on the literature-reported field experiment data and found that the models of C3 crops (wheat and rice) and C4 (maize) were both able to simulate the yield responses to doubled CO_2_ sufficiently close to reported data, especially after the re-calibration of underlying leaf-level assimilation processes used in the latest version of DSSAT, which was also used in this study. See Boote et al. (2011) for more details.

Crops response to CO_2_ in GGCMI models has been shown to produce optimistic results, as ozone concentrations, which are expected to increase, can have an opposite and potentially larger effect than elevated CO_2_ on plant productivity ^37,40,41^. The degree to which CO_2_-enhanced photosynthesis may result in higher crop yields is also unclear, considering the effects of competing plant physiological processes that down-regulate photosynthesis, confounding effects from nutrient limitation, and growth in plant tissues/organs other than the storage parts that are harvested ^42^, as well as the possibility of higher susceptibility to herbivory from invasive pests ^43^.

We explored the effects of CO_2_ fertilization in simulations that use adoption rule 1 and 2. As expected, the production gains for the three cereals are larger than those reported in the main text and so are the reductions in prices and in the number of people at risk of hunger. By accounting for CO_2_ fertilization global production of maize can increase by an additional 0.6% (over what simulated without CO_2_ fertilization), rice production by 1.3% and wheat production remains practically unchanged – 0.1%.

The contribution to GHG abatement is extremely uncertain and substantial work must be done before GHG results generated by DSSAT can be trusted. However, just by considering the reduction in the area needed to satisfy the demand for maize and rice once can evince that GHG would be likely not be higher than those reported in the main text. Results for rice, for example, show that the increased productivity can reduce area harvested up to 1.7 million hectares and this can lead to an additional yearly reduction in emissions estimated at 3.7 Million tons of CO_2_ e

While these results cannot be trusted because of the arguments listed above, they do not change the general results of this study: the use of CSA practices can increase production and reduce GHG emissions.

S6. About Rosegrant et al. adoptions rates

Rosegrant et al. 2014 ^6^, impose a limit to adoption based on expert opinion and on criteria that reflect key characteristics of a technology (Table S 6). The imposed upper bound effectively reduces the number of hectares that potentially transition to the alternative practices. For instance, if there were one hundred hectares on which no-till returns higher yields than current practices, only seventy hectares would be considered as adopting no-till in the simulation.

**S7. AWD scenario**

If one considers that $\Pi_{0}=P*Y_{0}-{TC}_{0}$ and $\Pi_{AWD}=P*Y_{AWD}-\left( {TC}_{0}-{TC}_{0}*X_{AWD} \right)$ where $\Pi$ indicates profit, $P$ prices, $Y$ yields, $TC$ total costs of production, the subscripts $0 \mathrm{and} AWD$ identify non-adoption and adoption of AWD practices, and $X$ the percent reduction in production costs due to adoption of AWD. In order for AWD to be adopted it must be that $P*Y_{0}-{TC}_{0}<P*Y_{AWD}-\left( {TC}_{0}-{TC}_{0}*X_{AWD} \right)$ which is equivalent to $Y_{0}-Y_{AWD}<\frac{{TC}_{0}*X_{AWD}}{P}$. Recalling that in the long-run total costs are equal to total revenues one can rewrite $\Delta Y<\frac{{P*Y}_{0}*X_{AWD}}{P}\equiv\frac{\Delta Y}{Y_{0}}<X_{AWD}$.

This means that that AWD will be adopted as long as the percent reduction in yields due to the adoption of AWD practices is less than the percent reduction in production costs. Irrigation costs represent from 3 to 36% of production costs ^44–46^ and AWD is reported to reduce irrigation cost up to 30% ^47,48^. Based on this information, it is assumed that the adoption of AWD can reduce production costs up to 9%. Therefore, given these assumptions as long as yields decrease less than 9% AWD is still be preferable to the current practices.

S8. Level of adoption of CSA technologies

The effects on total crop production and on prices are dependent on how widely CSA practices and technologies are adopted. The adoption rates of the CSA technologies for the two adoption scenarios are shown in Table S 7 (expressed as share of cropland on which technologies are adopted). As expected, adoption across cropland is lower when the two conditions of reduction of emission intensity and increase in yields are satisfied. Adoption seems to decrease more for maize and wheat than for rice indicating that for these crops the considered practices do not automatically lead to a reduction of emissions. CSA practices are adopted on a total of approximately 372 million hectares in the first scenario (top row) and 241 million hectares in the second (bottom row).

**S9. Interpretation of endogenous shifts in prices and area**

In the modeling framework, simulations of adoption of new technologies/practices, as well as shocks from climate change enter the global food market through changes in agricultural yields. Specifically, the biophysical changes in productivity enter the IMPACT model via its link with crop models. Ultimately, the core equations in IMPACT assess the aggregate effect of economic (income), population, climate scenarios and technology adoption on agriculture productivity, harvested areas, and food security by simulating how the global food market responds to the interactions among these drivers. The IMPACT baseline (BAU) estimates an increase in food prices between 2010 and 2050 because population and income growth, encoded in the SSP2 scenario, increase demand for food. Some of the increase in prices is also due to climate change shocks impacting cereals productivity, globally. As a global aggregate, CSA technologies lead to an increase of maize, rice and wheat yields, which results in a price reduction for the three crops compared to the BAU scenario without these technologies. In general, consumers respond to lower prices by increasing consumption of those commodities that became cheaper, especially when they constitute a staple of their diet. At the same time, lower market prices have some effects in discouraging net producers from further expanding production or investing in inputs (and therefore reinforcing yields) for those crops whose prices have fallen the most. The latter often translates into a decrease in harvested area, and into an endogenous feedback loop effect on yields. The global aggregate result on areas and yields also depend on which regions see the largest/lowest benefits from CSA vis-à-vis their role and contribute to both global production and trade of those crops. (e.g. how large of a producer the region is, and whether it is a net exporter/importer and how large their role is in trade). Finally, yields and area results determine total agricultural production. Taking into consideration population and GDP growth, the resulting change in food availability from CSA may translate into a larger availability of kilocalories (Robinson et al. 2015) in some regions, and into a global increase in food security. In IMPACT, the share of people at risk of hunger (or at risk of food insecurity) is calculated based on an empirical correlation between the share of undernourished people within the population and the relative availability of food. The calculation is adapted from Fischer et al. (2005) (Robinson et al. 2015). The share of undernourished children under the age of five is based on the calculation of the average calorie availability per capita per day, women’s access to secondary education, the ratio of female to male life expectancy at birth, and health and sanitation (Smith and Haddad 2000; Robinson et al., 2015).

S10. Emissions intensity, yields and area

Total emissions from crop production $\left( E \right)$ are determined by a multiplicative combination of emission intensity (*e*, emissions per unit of output), yields (*y*, output per hectare), and area (*a*, hectares allocated to crop production):

$\boldsymbol{E=f}\left( \boldsymbol{e, y, a} \right)\boldsymbol{=e*y*a}$ Eq.1

Eq.1 indicates that emission reductions depend on the effectiveness of a practice or a technology to reduce emissions per unit of output (emission intensity) but that it is possible for yields and area to increase sufficiently to offset reduction in emission intensity (as shown for certain countries in Table S 11). This can be easily observed noting that an approximation of the changes total emissions from initial levels of intensity, yield and area $\left( e^{*};y^{*};a^{*} \right)$ can be obtained by taking the total derivative of equation 1,

$dE=\frac{\partial f}{\partial e}\left( e^{*} \right)de+\frac{\partial f}{\partial y}\left( y^{*} \right)dy+\frac{\partial f}{\partial a}\left( a^{*} \right)da$,

and by noting that $\frac{\partial f}{\partial e}, \frac{\partial f}{\partial y},\mathrm{and} \frac{\partial f}{\partial a}are all>0$.

S11. GHG emissions – Area reallocation

Consider a program that targets a group of crops $J$ for the adoption of CSA practices we can divide land area in two components, land allocated to the $J$ crops that are the target of intervention (adoption of CSA practices) and land allocated to all other agricultural uses (identified with the superscript $O$ in the equations that follow). Total emissions at any time $t$ will therefore be:

$E_{t}=\sum_{O} e_{t}^{o}y_{t}^{o}a_{t}^{o}+\sum_{j} e_{t}^{j}y_{t}^{j}a_{t}^{j}$

Where *e* is emissions per unit of output, *y* is yields (output per hectare), and *a* is area (hectares allocated to crop production).

Let us consider two time periods and two possible development paths, one in which there is no adoption of CSA technologies, the baseline indicated with the superscript $B$, and one with adoption, indicated by superscript *A*. The first time period $\left( t_{1} \right)$, is common to both scenarios and indicates the initial conditions. During the second time period $\left( t_{2} \right)$ it is possible for farmers to adopt CSA technologies. For the baseline, the change in emissions between the two time periods is:

$$E_{t_{1}}-E_{t_{2}}^{B}=\sum_{O} e_{t_{1}}^{o}y_{t_{1}}^{o}a_{t_{1}}^{o}+\sum_{j} e_{t_{1}}^{j}y_{t_{1}}^{j}a_{t_{1}}^{j}-\sum_{O} {e_{t_{2}}^{o}}^{B}{y_{t_{2}}^{o}}^{B}{a_{t_{2}}^{o}}^{B}-\sum_{j} {e_{t_{2}}^{j}}^{B}{y_{t_{2}}^{j}}^{B}{a_{t_{2}}^{j}}^{B}$$

While for the scenario with adoption, the change in emissions is:

$$E_{t1}-E_{t2}^{A}=\sum_{O} e_{t_{1}}^{o}y_{t_{1}}^{o}a_{t_{1}}^{o}+\sum_{j} e_{t_{1}}^{j}y_{t_{1}}^{j}a_{t_{1}}^{j}-\sum_{O} {e_{t_{2}}^{o}}^{A}{y_{t_{2}}^{o}}^{A}{a_{t_{2}}^{o}}^{A}-\sum_{j} {e_{t_{2}}^{j}}^{A}{y_{t_{2}}^{j}}^{A}{a_{t_{2}}^{j}}^{A}$$

For reasons of generality, we assume that farmers adopt CSA technologies only on a fraction $p$ of the area where target crops are grown

$$E_{t1}-E_{t2}^{A}=\sum_{O} e_{t_{1}}^{o}y_{t_{1}}^{o}a_{t_{1}}^{o}+\sum_{j} e_{t_{1}}^{j}y_{t_{1}}^{j}a_{t_{1}}^{j}-\sum_{O} {e_{t_{2}}^{o}}^{A}{y_{t_{2}}^{o}}^{A}{a_{t_{2}}^{o}}^{A}-\sum_{j} {e_{t_{2}}^{j}}^{A}{y_{t_{2}}^{j}}^{A}p^{j}{a_{t_{2}}^{j}}^{A}-\sum_{j} {e_{t_{2}}^{j}}^{A}{y_{t_{2}}^{j}}^{A}d^{j}{a_{t_{2}}^{j}}^{A}$$

where $d=\left( 1-p \right)$.

Emissions from crop production will decrease when the following condition is satisfied:

$$E_{t1}-E_{t2}^{B}-\left( E_{t1}-E_{t2}^{A} \right)<0$$

Noting that for the portion $d$ of the crop $j$ in which the CSA practices are not adopted ${e_{t_{2}}^{j}}^{A}{y_{t_{2}}^{j}}^{A}={e_{t_{2}}^{j}}^{B}{y_{t_{2}}^{j}}^{B}$and assuming that emission intensity and yields experience the same change through time when CSA practices are not adopted, it follows ${e_{t_{2}}^{o}}^{A}{y_{t_{2}}^{o}}^{A}={e_{t_{2}}^{o}}^{B}{y_{t_{2}}^{o}}^{B}$, after some simple algebraic manipulations we obtain:

$\underset{\boldsymbol{Change in emissions due to adoption of CSA}}{\underbrace{\sum_{j} {e_{t_{2}}^{j}}^{B}{y_{t_{2}}^{j}}^{B}p^{j}{a_{t_{2}}^{j}}^{B}-\sum_{j} {e_{t_{2}}^{j}}^{A}{y_{t_{2}}^{j}}^{A}p^{j}{a_{t_{2}}^{j}}^{A}}}>\underset{\boldsymbol{Change in emissions due to changes in area in all other uses}}{\underbrace{\sum_{O} {e_{t_{2}}^{o}}^{B}{y_{t_{2}}^{o}}^{B}*\sum_{O} {a_{t_{2}}^{o}}^{A}-{a_{t_{2}}^{o}}^{B}}}+\underset{\boldsymbol{Change in emissions due to changes in area in target crops not adopting CSA}}{\underbrace{\sum_{j} {e_{t_{2}}^{j}}^{B}{y_{t_{2}}^{j}}^{B}*\sum_{J} d^{j}\left( {a_{t_{2}}^{j}}^{A}-{a_{t_{2}}^{j}}^{B} \right)}}$Eq. 2

Some additional considerations are necessary regarding changes in GHG emissions. Equation 2 indicates that reducing emissions through the adoption of CSA practices is an important condition for a reduction in total emissions, but it is not sufficient and that changes in emissions on areas allocated to all other land uses must be also be considered. This is because changes in world prices can affect all land-uses through changes in profitability at the local level but also through change in countries’ comparative advantage in producing agricultural commodities. The IMPACT model only considers cropland in its calculations for the supply of agricultural commodities and therefore statements regarding changes in other land uses such as forested or pasture areas cannot be made. Staying within the limits of the modeling environment, we can however note that while harvested area for rice and wheat is reduced by a total of 10 to 13 million hectares, area allocated to other crops increases, essentially replacing wheat and rice. In particular, soybeans, vegetables, other minor crops, temperate fruits and sugarcane are the most affected with a total increase of more than two million hectares Table S 13. Depending on how they are grown, these crops might have a higher carbon footprint than the crops they replace in the field and therefore partially offset the reduction in GHG emissions generated by the adoption of CSA alternative practices.

It is important to note that even though globally GHG are reduced in both scenarios, due to rearranging of production areas and increases in yields, there are countries for which emissions are estimated to increase, as already previously shown in Table S 11. This holds true even in the scenario in which a reduction of emission intensity is required. This finding confirms, as equation 1 shows, that reducing emission intensity is not a sufficient condition to reduce total emissions.

S12. Indirect effects on GHG emissions – livestock

The modeling setting used in this study does not allow for a life-cycle analysis of GHG emissions nor the analysis of the changes occurring to other economic sectors beside the agricultural sector. It is possible however to investigate other possible sources of GHG emissions affected by the reorganization of the agricultural markets induced by the sizable effects of increased production. For example, it is possible to examine the effects on cattle production, an important source of GHG emissions. Emissions from enteric fermentation and manure are estimated to be about 3.1 Gt per year, about 6 % of all anthropogenic GHG emissions ^27^. Of these, 65% are attributed to cattle raised for both beef and milk ^49^.

IMPACT simulations show that growth in population and in countries’ GDP are such that the global demand for beef may increase by more than 75 % between 2010 and 2050, and the demand of milk by 60 %, irrespective of the climate scenario. Increased production in maize, wheat, and rice increases the availability of livestock feed, reduces its price, and makes it possible to increase the number of animals that can be supported globally.

Livestock, and specifically ruminants (e.g., cows, buffaloes, camels, and goats) are important sources of CH4 ^27^. IMPACT projects the number of dairy animals, and that of slaughtered cattle (because the model is interested in meat, as a commodity). We estimated CH4 emissions from ruminants based on the total number of animals and on emission factors from the enteric fermentation section of FAOSTAT (http://www.fao.org/faostat/en/#data/GE).

For non-dairy animals, we estimated the global herd size by first calculating the ratio global herd (FAO, non-dairy cows) by slaughtered animals (IMPACT) for the year 2010 (the ratio is 4.4). This value was then multiplied by the IMPACT-projected slaughtered animals for 2050. To calculate total emissions in 2050, the per-head emissions from enteric fermentation obtained from FAOstat was multiplied by the computed global herd.

For dairy animals, we used FAOSTAT data on emissions per head for dairy animals listed in the enteric fermentations section. The emission factor was then multiplied by the projected number of milk-producing animals in 2050.

Future livestock management strategies, including changes in feed composition are likely to change the emissions per head. Modeling these changes are beyond the scope of the present study, therefore we worked under an assumption of constant emissions per-head.

The indirect effects on the cattle industry are such that emissions from enteric fermentation increase by approximately 3 - 6 Mt yr-1 CO2e by 2050 (Table S 14). While the productivity gains described earlier suggested a reduced encroachment of crop land into other land uses, these additional animals would require additional pastureland and offset in part the positive effects of reduced for land expansion.

S13. Adaptation effects of CSA practices

Using IMPACT, it is possible to isolate the effects of climate change on the agriculture sector. By creating a scenario that uses the current weather parameters (climate around the year 2005) and retains all the other assumptions on population and GDP growth and comparing the results of a scenario so constructed with one that uses GCMs projections about future weather, one can assess climate change-related effects on global production and global prices ^5,18^. Figure S 8 includes a No-climate change (NoCC) scenario and shows how globally climate change represented by the BAU scenario negatively effects maize and rice production with resulting increases in prices. The situation for wheat is different with global wheat production projected to increase with climate change. This is the result of higher production at higher latitudes and greater area allocated to wheat due to increased demand for wheat products. CSA practices are projected to offset the negative effects on maize and rice production and slow down consequent increases in prices. Similarly, CSA practices reduce the growth in wheat price which is projected to increase even in presence of higher global production.

**Supplementary** **figures**

Figure S 1 The IMPACT system of models. Modified from Robinson et al. 2015

| 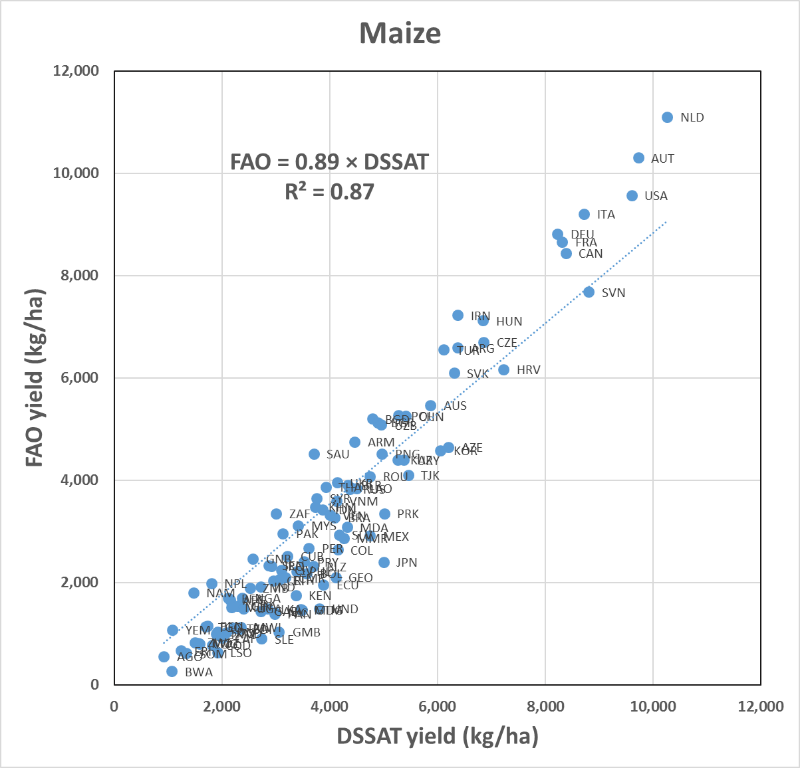 |
| --- |
| 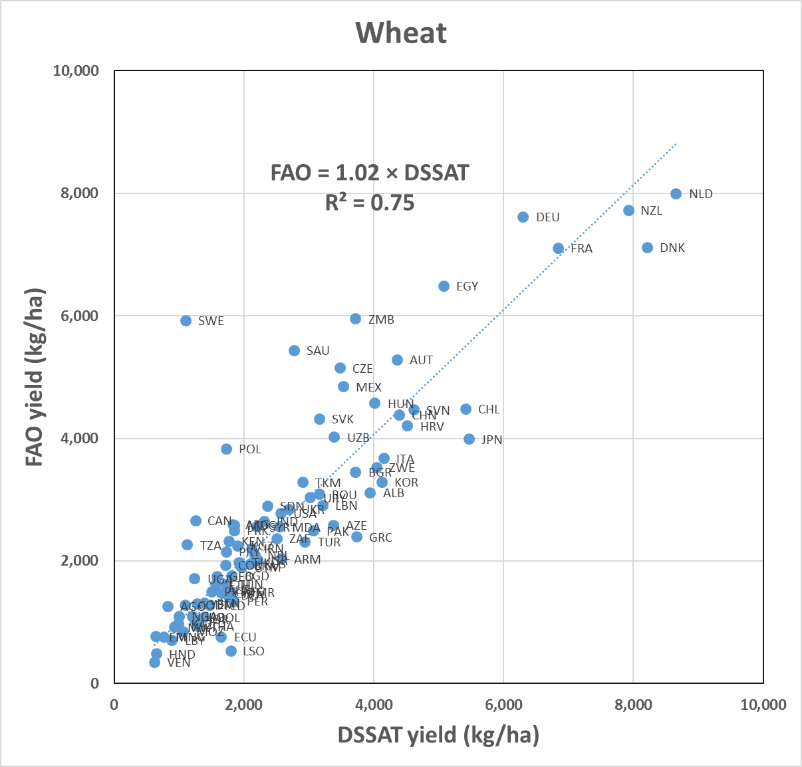 |
| 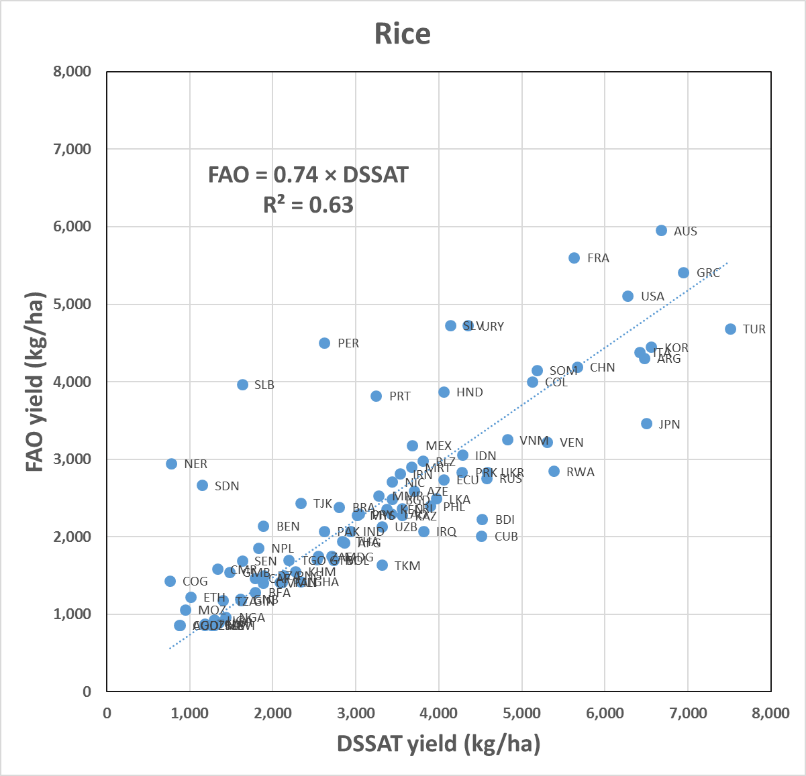 |

Figure S 2 DSSAT calibration results summarized for each country in the World

Source: Authors

Notes: DSSAT= Decision Support System for Agrotechnology Transfer model; FAO= Food and Agriculture Organization of the United Nations


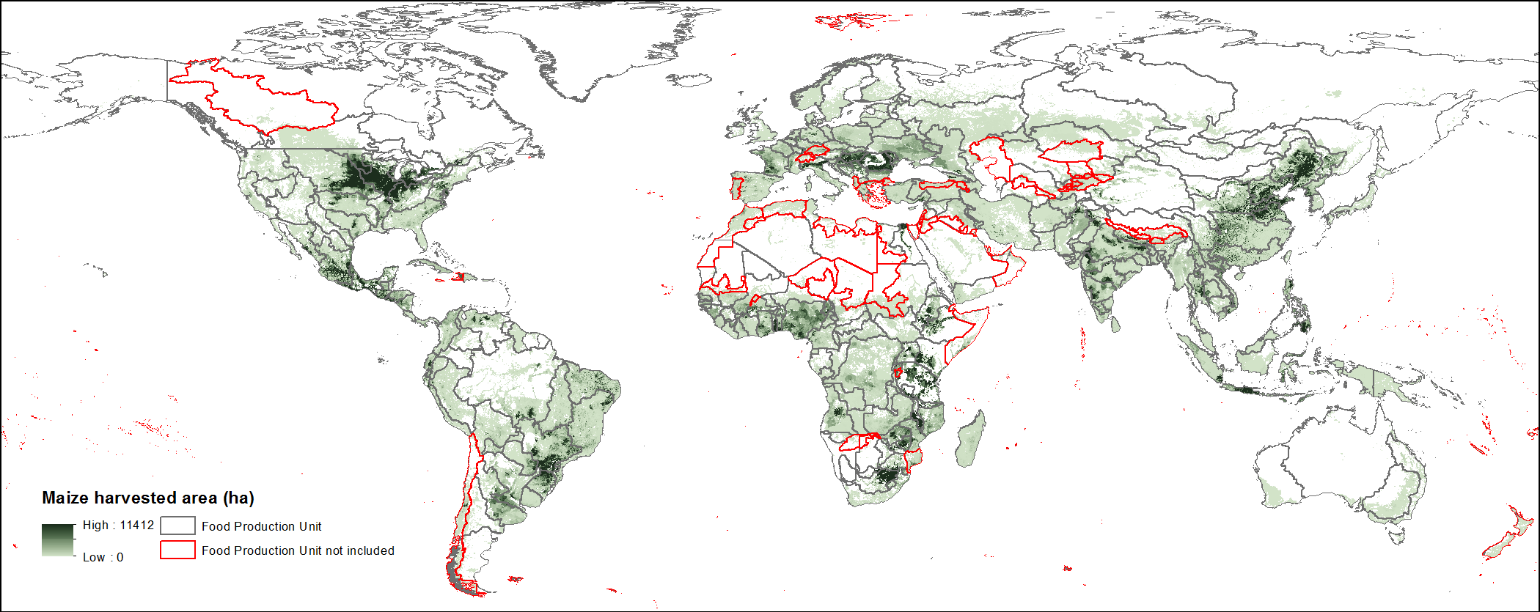


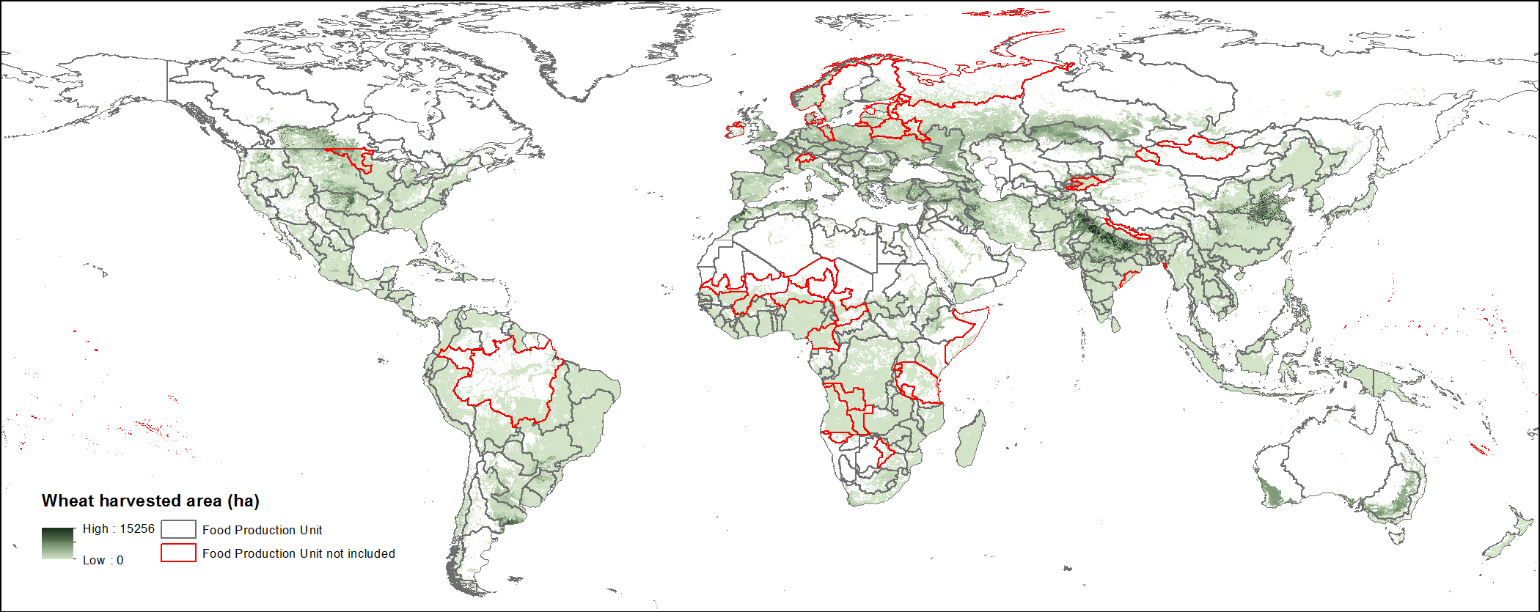


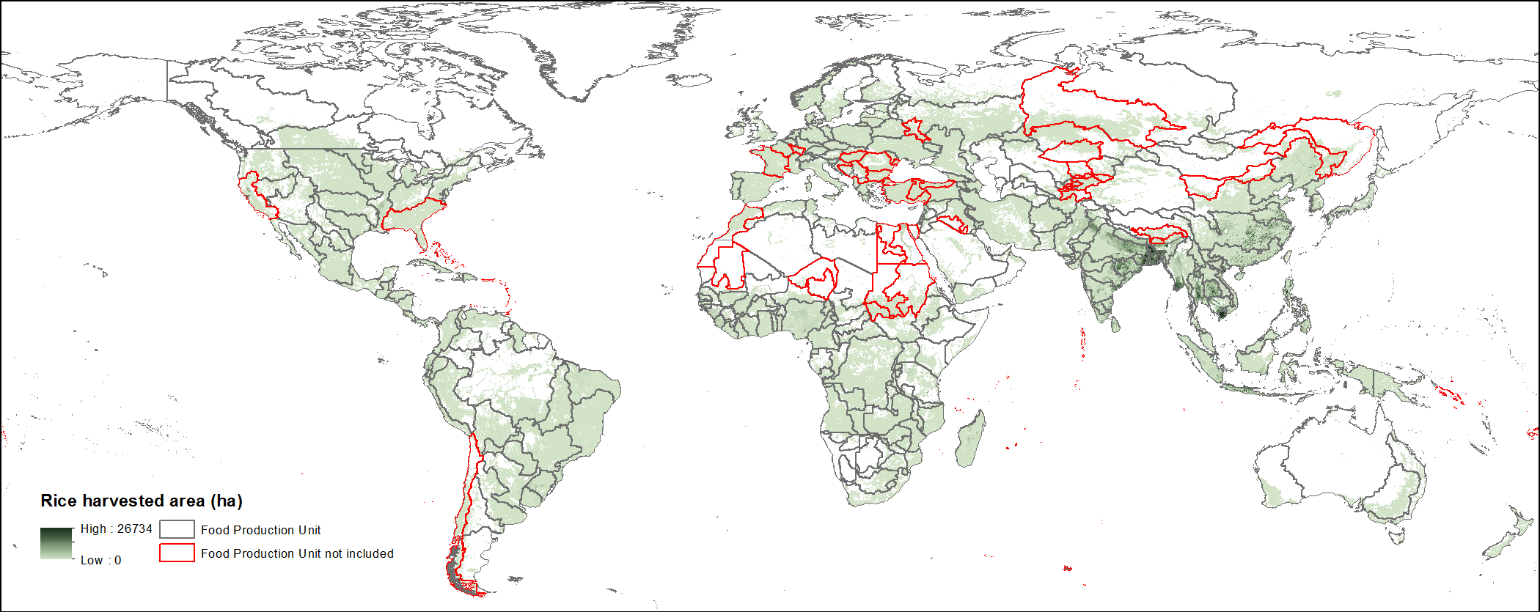


Figure S 3. Distribution of cropland across IMPACT FPUs. Red line indicates FPUs not included in this modeling after DSSAT calibration.

Source: Authors

| GFDL-ESM2M |
| --- |
| 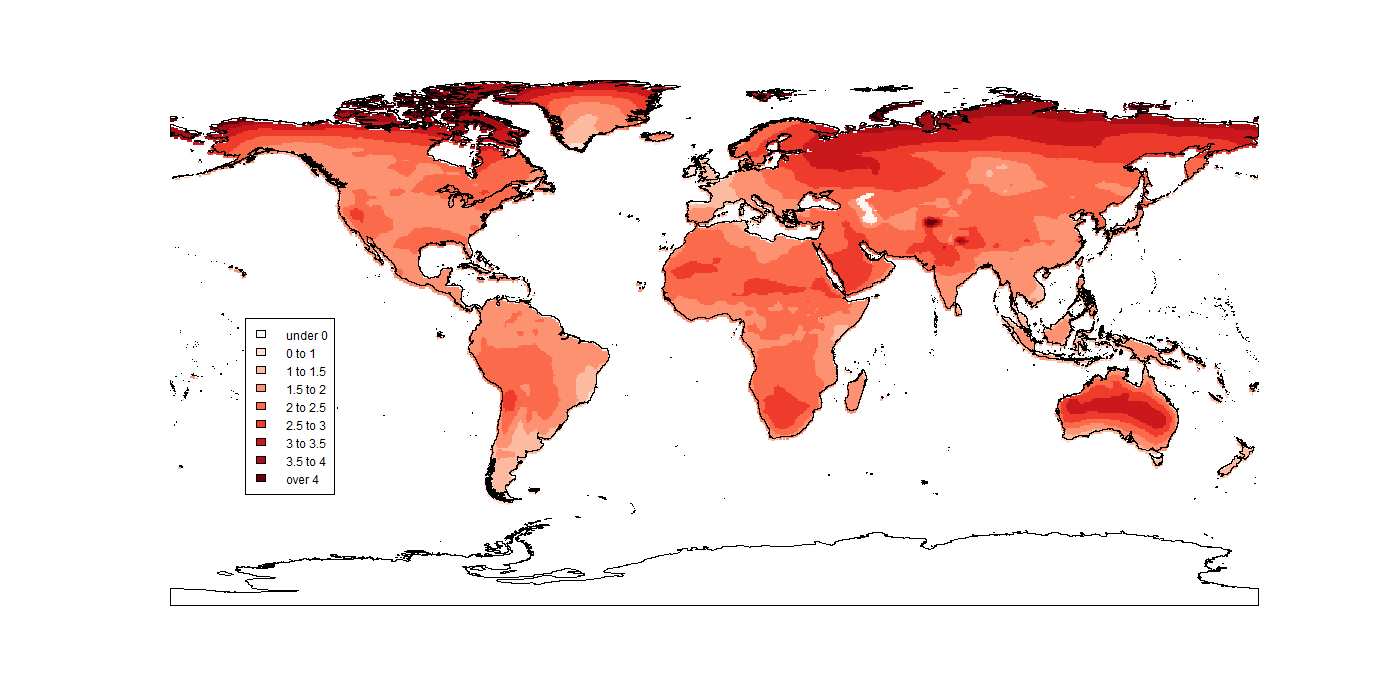 |
|  |
|  |
| HadGEM2-ES |
| 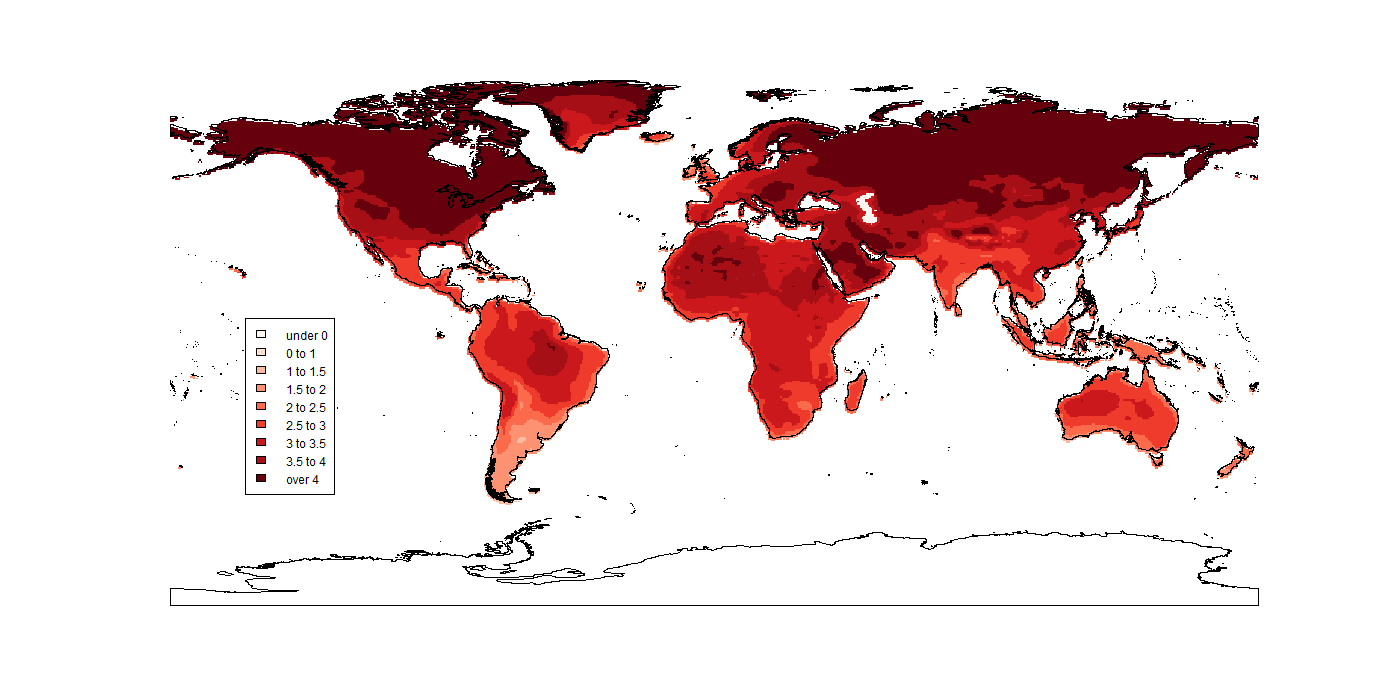  Difference in temperature between GFDL and HadGEM |
| 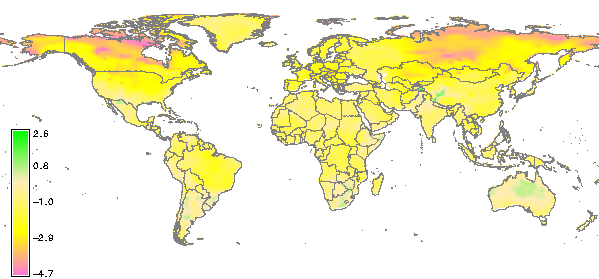 |

Figure S 4 Changes in maximum temperature in 2050 compared with 2000 (degrees °C), and difference among the two earth system models (GFDL minus HadGEM). Representative concentration pathway (RCP) 8.5. Adapted from Cenacchi et al. 2016 ^50^

Source: Authors

| GFDL-ESM2M |
| --- |
| 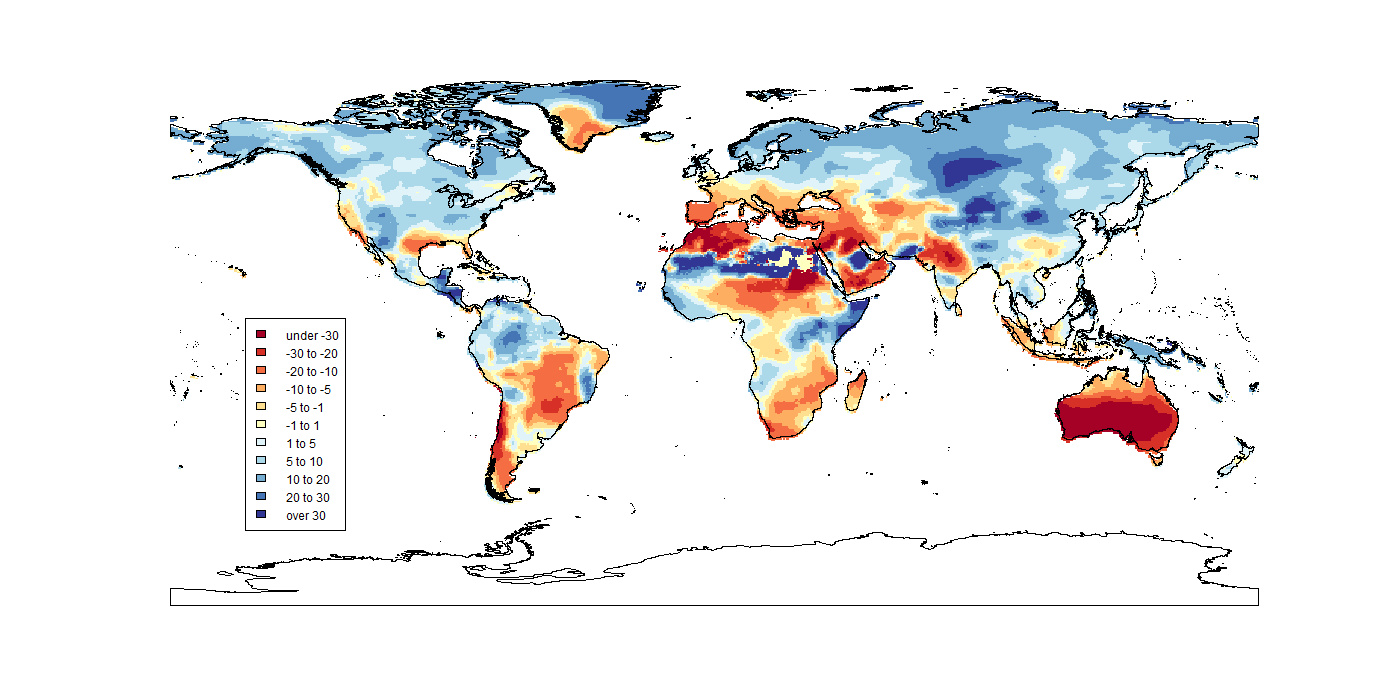 |
|  |
|  |
| HadGEM2-ES |
| 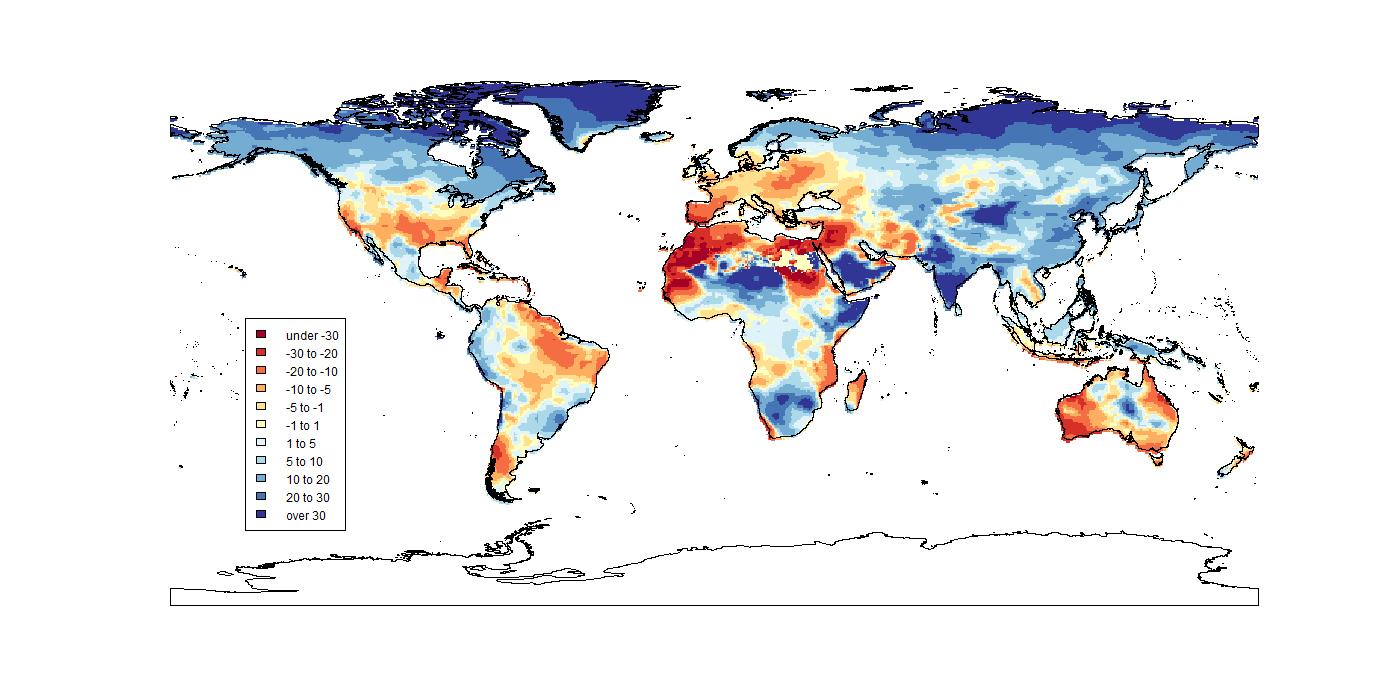  Difference in precipitation between GFDL and HadGEM  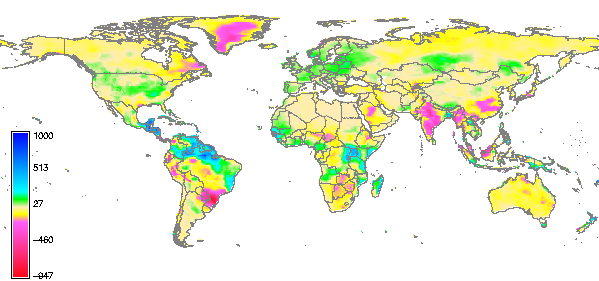 |

Figure S 5 Changes in annual precipitation in 2050 compared with 2000 (millimeters of rain) and differences among the two earth system models (GFDL minus HadGEM). Representative concentration pathway (RCP) 8.5. Adapted from Cenacchi et al. 2016 ^50^

Source: Authors

| Rule 1  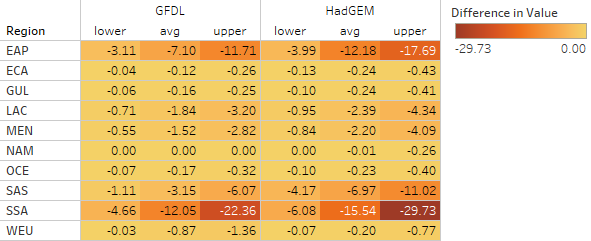 |
| --- |
| Rule 2  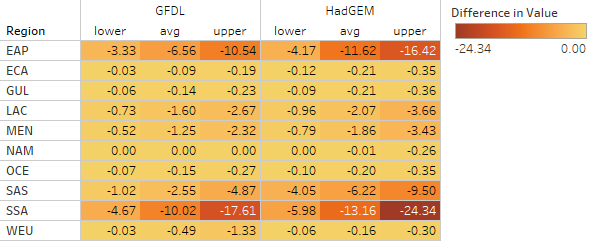 |

Figure S 6 Population at risk of hunger. Difference between CSA adoption Rule 1 and Rule 2 and BAU in 2050 (million people). Negative values indicate a decrease compared to BAU

Source: Authors

| Rule 1  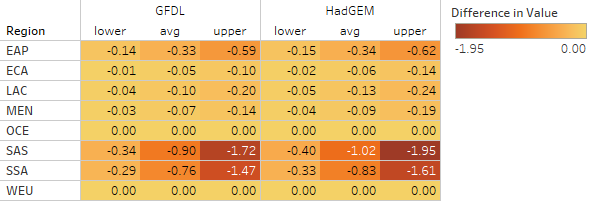 |
| --- |
| Rule 2  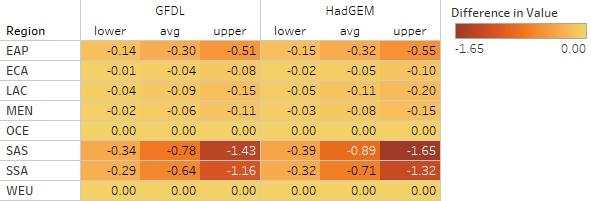 |

Figure S 7. Undernourished children (age 0-5). Difference between CSA adoption Rule 1 and Rule 2 and BAU in 2s050 (million children). Negative values indicate a decrease compared to BAU

Source: Authors

| 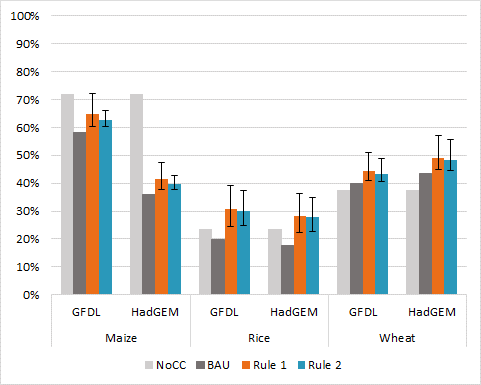  Production | 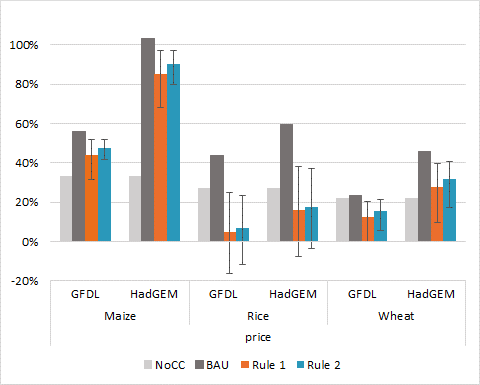  Price |
| --- | --- |

Figure S 8. IMPACT simulations. Changes in production and prices between 2010 and 2050. NoCC: baseline scenario without climate change effects; BAU: baseline scenario with climate change

Source: Authors

**Supplementary** **Tables**

Table S 1. DSSAT input and output data

| INPUT | OUTPUT |
| --- | --- |
| **Site Information**  Coordinates, elevation, drainage | **Phenology**  Flowering, gran/seed/tuber, maturity |
| **Daily weather**  Solar radiation, temperature (max,min), precipitation | **Yield component**  Grain/seed, biomass, LAI |
| **Soil**  Classification, water release curve, bulk density, organic carbon, acidity, root growth factor, drainage coefficient | **Soil**  nitrogen balance (e.g., leaching) water balance (e.g., runoff) carbon balance (e.g., emission) phosphorus balance |
| **Initial conditions**  previous crop, soil water and nitrogen content |  |
| **Management**  cultivar, planting, water and nutrient management, residue application, tillage, harvest, pest/disease damage |  |

Source: Authors

Table S 2 minimum yields used in DSSAT calibration

| Crop | Water Management | Yield (dry matter Mg ha^-1^) |
| --- | --- | --- |
| Wheat | Irrigated | 0.35 |
| Wheat | Rainfed | 0.35 |
| Maize | Irrigated | 0.61 |
| Maize | Rainfed | 0.26 |
| Rice | Irrigated | 0.95 |
| Rice | Rainfed | 0.65 |

Source: Authors

Table S 3 Comparison of literature based on field experiments with simulated results. The latter is statistically summarized by aggregating outputs, which are obtained from the first 10 yrs of simulations at each grid cell, for each country based on ISO3 code. Note that values reported in literature might not have all statistics (e.g. median, minimum, or maximum).

| **Yields** | | | | | | | |
| --- | --- | --- | --- | --- | --- | --- | --- |
| **Crop** | **Technology** | **Source** | **Region** |  | | **Changes in yields in %** | |
|  |  |  |  |  |  | Median reported  (min, max) | Median  Simulated  (min, max) |
| Rice | AWD | Huda et al. (2016) ^51^ | Bangladesh |  |  | 0 ** | -22 (-54, 83) |
| Rice | AWD | Pandey et al. (2014) ^52^ | Vietnam |  |  | - 5.7 ** | -18 (-40, 15) |
| Maize | isfm | Chivenge et al. (2009) ^53^ | Kenya |  |  | 32 (-20, 40) | 40 (-6.2, 140) |
| Wheat | isfm | Agegnehu et al. (2014) ^54^ | Ethiopia |  |  | (68, 129)* | 6 (-0.2, 51) |
| Wheat | no till | Erenstein (2009) ^55^ | Indo-Gangetic plains |  |  | (5, 7)* | 11 (-11, 221) |
| Maize/wheat | no till | Hobbs et al. (2008); Govaerts et al. (2005) ^24,56^ | Mexico |  |  | (8, 30)* | 0.8 (-16, 145) |
| Maize | no till | Ito et al. (2007) ^57^ | Ethiopia |  |  | 10 ** | -7.5 (-31, 184) |
| Wheat | no till | Ito et al. (2007) | Ethiopia |  |  | 9 ** | 0 (-12, 9) |
| Maize | no till | Ito et al. (2007) | Malawi |  |  | -1.5 (avg) ** | -3.8 (-10, 5.4) |
| Maize | no till | Ito et al. (2007) | Mozambique |  |  | 270 (avg) ** | -3.8 (-8.9, 29) |
| Rice | UDP^1^ | Huda et al. (2016) (35) | Bangladesh |  | | (29, 40)* | 5 (-36, 119) |
| Rice | UDP^1^ | Bandaogo et al. (2015) ^58^ | Burkina Faso |  |  | (5, 12)* | -17 (-48, 169) |
| **CO_2_** | | | | | | | |
| **Crop** | **Technology** | **Source** | **Region** | **SOC accumulation in kg C ha^-1^** | |  | |
|  |  |  |  | Median Obs (min, max) | Median Sim  (min, max) |  |  |
| Wheat | Noti | Powlson et al. (2014) ^59^ | indo-gangetic plains | 300 ** | 30 (-155, 157) |  |  |
| All crops | Noti | Denef et al. (2011); Ogle et al. (2005, 2010); Six et al. (2004); West and Marland (2002) ^60–64^ | USA | (10, 700)* | 40 (-521, 560) |  |  |
| All crops | isfm | Denef et al. (2011) (45) | USA | 400 ** | 25 (-199, 624) |  |  |
| Maize | no till | Beheydt et al. (2008) ^65^ | Belgium | 600 ** | 114 (-112, 308) |  |  |
| **CH_4_** | | | | | | | |
| **Crop** | **Technology** | **Source** | **Region** | **Emission in kg CH4 ha^-1^** | | **Changes of Emission in %** | |
|  |  |  |  | Median Obs (min, max) | Median Sim  (min, max) | Median Obs (min, max) | Median Sim  (min, max) |
| Rice | Base | Wang et al (1993); Wang M. (1995); Lu et al. (1995) ^66–68^ | China | 340 (50, 1,550) | 325 (63, 902) |  |  |
| Rice | Base | Adhya et al. (1994) ^69^ | India | 200 (50, 300) | 300 (77, 1,133) |  |  |
| Rice | Base | Nugroho et al (1994) ^70^ | Indonesia | 310 (140, 470) | 100 (74, 465) |  |  |
| Rice | Base | Holzapfel-Pschorn & Seiler  1986; Schütz et al. 1989 ^71,72^ | Italy | (120, 770)* | 241 (140, 1,069) |  |  |
| Rice | Base | Shin Y. K. et al 1995; ^73^ | Korea | 330 (90, 630) | 279 (84, 409) |  |  |
|  |  | Metra-C.et al.1995 ^74^ | Philippines | 270 (100, 870) | 98 (72, 3,578) |  |  |
| Rice | Base | Murase et al. 1994 ^75^ | Thailand | 480 (340, 860) | 313 (78, 471) |  |  |
| Rice | Base | Lindau et al 1991;  Cicerone et al, 1992;  Sass & Fisher 1995 ^76–78^ | USA | 250 (10, 480) | 530 (373, 650) |  |  |
| Rice | AWD | Adhya et al. (2014) ^79^ | USA |  |  | (-45, -90)* | -57 (-50, -66) |
|  |  |  | Tamil Nadhu and Punjab (India) |  |  | (-30, -80)* | -48 (-35, -55) |
|  |  |  | Philippines |  |  | (-10, -80)* | -48 (-46, -50) |
| Rice | AWD | Pandey et al. (2014) ^52^ | Vietnam |  |  | (-250, )* | -49 (-45, -53) |
| **N_2_O** | | | | | | | |
| **Crop** | **Technology** | **Source** | **Region** | **Emission in kg N_2_O-N ha^-1^** | | **Changes of Emission in %** | |
|  |  |  |  | Median Obs (min, max) | Median Sim  (min, max) | Median Obs (min, max) | Median Sim  (min, max) |
| Rice | Base | Cai et al (1999); Xiong et al (2002); Khalil et al (1998); Zheng et al (2000) ^80–83^ | China | (0.1, 4.4)* | 1.2 (0.1, 20) |  |  |
| Rice | Base | Kumar et al (2000); Majumdar et al (2000); Pathak et al (2002) | India | (0.03, 0.9)* | 1.6 (0, 14) |  |  |
| Rice | Base | Suratno et al (1998) ^84–87^ | Indonesia | (0.3, 1.1)* | 1.5 (0, 14) |  |  |
| Rice | Base | Yagi et al (1996) ^88^ | Japan | (0.03, 0.07)* | 1.1 (0.2, 12) |  |  |
| Rice | Base | Bronson et al (1997) ^89^ | Philippines | (0.06, 0.6)* | 1.4 (0, 8) |  |  |
| Rice | AWD | Pandey et al. 2014; Dong et al. 2012 ^52,90^ | Vietnam |  |  | (139, 600)* | 890 (166, 2300) |
| Rice | UDP^a^ | Gaihre et al. 2015 ^91^ | Bangladesh |  |  | (-84, -61)* | 14 (-93, 406) |
| maize | isfm | Muhammad et al. 2011 ^92^ | field experiment (Australia) |  |  | (34, 42)* | -57 (-98, 11) |
| All crops | isfm | Frimpong et al. 2010 ^93^ | microcosm experiment Ghana |  |  | (-8, 270)* | 18 (-5.4, 314) |
| **Water saving** | | | | | | | |
| **Crop** | **Technology** | **Source** | **Region** |  | | **Water saving in %** | |
|  |  |  |  |  |  | Median Obs (min, max) | Median Sim  (min, max) |
| Rice | AWD | Lampayan et al. 2003; Palis et al. 2004; Rejesus et al. 2011; Lampayan 2013 and 2015 ^94–98^ | Philippines |  |  | (5, 50)* | 32 (26, 47) |
| Rice | AWD | Singh et al. 1996 ^99^ | India |  |  | (40, 70)* | 1. 3.5, 73) |

Source: Authors

Notes:

^a^ UDP in our simulations uses 70% of N fertilizer rates applied to business-as-usual.

* Median was not reported in these studies

** No range available in these studies

Table S 4 GWP conversions

| GHG | From Units | To Units | Conversion | GWP based on AR5 |
| --- | --- | --- | --- | --- |
| dSOC (net CO_2_ sequestration) | kgC | kgCO2eq | kgC * (-1 * 44 / 12)*GWP | 1 |
| CH_4_ | kgC | kgCO2eq | kgC * (16/12)*GWP | 28 |
| N_2_O | kgN | kgCO2eq | kgN * (44/28)*GWP | 265 |

Source: Authors based on Myhre et al. 2013 ^100^

Table S 5 shows the annual difference in average global precipitation and maximum and minimum temperatures between a reference climate, representative of conditions around the year 2000, and two climate change scenarios obtained by running the GFDL, and HadGEM earth system models under RCP 8.5. Data are for the year 2050.

|  | ∆ total precipitation (mm/year) | |  | ∆ average temperature maximum (^o^C) | |  | ∆ average temperature minimum (^o^C) | |
| --- | --- | --- | --- | --- | --- | --- | --- | --- |
|  | GFDL | HadGEM |  | GFDL | HadGEM |  | GFDL | HadGEM |
| Agricultural Area | 1.1 | 44.8 |  | 2.0 | 3.3 |  | 1.9 | 3.2 |
| Land Area | 13.9 | 29.4 |  | 2.1 | 3.6 |  | 2.1 | 3.7 |

Source*:* Authors.

Notes*:* a) The data area shown for values either over the agricultural land, or across the total land of the country. GFDL = Geophysical Fluid Dynamic Laboratory; HadGEM = Hadley Centre’s Global Environment Model; b) RCP data was downloaded from the RCP Database, version 2.0.5^101^ ; the data downloaded was for RCP 8.5 ^102^. To represent some of the uncertainty inherent in climate change projections, we use two climate change scenarios. The scenarios are based on results from running two climate models under a Representative Concentration Pathway (RCP) of 8.5 watts/m^2^ ^103^, each of which is combined with the IPCC’s “middle of the road” GDP and population growth scenario (Shared Socioeconomic Pathway 2, or SSP2) ^104^.

The two climate models, or Earth System Models (ESMs) as defined by IPCC AR5, are as follows:

- GFDL-ESM2M ^105^—designed and maintained by the US National Oceanic and Atmospheric Administration’s Geophysical Fluid Dynamic Laboratory (GFDL) ([www.gfdl.noaa.gov/earth-system-model](http://www.gfdl.noaa.gov/earth-system-model))
- HadGEM2-ES ^106^—the Hadley Centre’s Global Environment Model, version 2 ([www.metoffice.gov.uk/research/modelling-systems/unified-model/climate-models/hadgem2](http://www.metoffice.gov.uk/research/modelling-systems/unified-model/climate-models/hadgem2))^[[1]](#footnote-1)^

Table S 6 Assumed maximum level of adoption by technology (from Rosegrant et al.)

| CSA technology | Adoption ceiling (%) |
| --- | --- |
| No tillage (NT) | 70 |
| Integrated soil fertility management (ISFM) | 40 |
| Alternative wetting and drying (AWD) | 40 |
| Urea deep placement (UDP) | 40 |

Source: From Rosegrant et al. (2014) ^6^

Table S 7 Global adoption rate by crop under the different climate and adoption scenarios. The numbers represent the share of total cropland (for each of the three crops) under CSA in 2050. In each case the rest of the area is under the BAU technologies. Data refer to the average CSA tailoring scenario.

| Scenario | Adoption rate of CSA practice  Maize  (GFDL – HADGEM) | Adoption rate of CSA practice  Wheat  (GFDL – HADGEM) | Adoption rate of CSA practice  Rice  (GFDL – HADGEM) |
| --- | --- | --- | --- |
| Rule 1: Adoption of CSA practices dependent on increased yields | 70.2 %– 72.9% | 73.9% – 75.3% | 51.0% – 55.9% |
| Rule 2: Adoption of CSA practices dependent on reduction of emission intensity *and* increased yields | 37.8% – 38.8% | 45.0% – 47.8% | 42.8% – 47.3% |

Source: Authors

CSA= climate smart agriculture

Table S 8 Changes in areas and production by country and scenario

Tables available as an excel file in the GitHub repository: Changes in_Prod_and_Area_CTY_allscenarios.xlsx

Table S 9 Percent change in price of crops in 2050 compared to BAU under the different climate and adoption scenarios.

| Scenario | CSA Tailoring | Maize  GFDL – HADGEM | | Wheat  GFDL – HADGEM | | | Rice  GFDL – HADGEM | | |
| --- | --- | --- | --- | --- | --- | --- | --- | --- | --- |
| Rule 1: Adoption of CSA practices dependent on increased yields | Lower | -2.6% | -3.0% | | -2.6% | -4.1% | | -12.9% | -13.4% |
|  | Average | -7.7% | -8.8% | | -9.1% | -12.3% | | -27.1% | -27.5% |
|  | Optimal | -15.5% | -17.2% | | -19.3% | -24.9% | | -41.6% | -42.1% |
| Rule 2: Adoption of CSA practices dependent on reduction of emission intensity *and* increased yields | Lower | -2.5% | -2.9% | | -2.0% | -3.4% | | -14.1% | -14.3% |
|  | Average | -5.3% | -6.3% | | -6.6% | -9.6% | | -25.8% | -26.4% |
|  | Optimal | -9.2% | -11.4% | | -14.5% | -19.6% | | -38.7% | -39.8% |

Source: Authors

Table S 10 Percent change and number of individuals in global population at risk of hunger and undernourished children (weight for age) in 2050 compared to BAU under the different climate and adoption scenarios

| Scenario | CSA Tailoring | Population at risk of hunger  GFDL – HADGEM | | Undernourished children  GFDL – HADGEM | |
| --- | --- | --- | --- | --- | --- |
| Rule 1: Adoption of CSA practices dependent on increased yields | Lower | -2.4%  (-10.3 Million) | -3.5%  (-16.4 Million) | -0.8%  (-0.8 Million) | -0.9%  (-1.0 Million) |
|  | Average | -6.3%  (-27. Million) | -8.4%  (-40.2 Million) | -2.2%  (-2.2 Million) | -2.4%  (-2.5 Million) |
|  | Optimal | -11.3%  (-48.5 Million) | -14.5%  (-69.1 Million) | -4.1%  (-4.2 Million) | -4.5%  (-4.7 Million) |
| Rule 2: Adoption of CSA practices dependent on reduction of emission intensity *and* increased yields | Lower | -2.4%  (-10.5 Million) | -3.4%  (-16.3 Million) | -0.8%  (-0.9 Million | -0.9%  (-1.0 Million) |
|  | Average | -5.3%  (-22.8 Million) | -7.5%  (-35.7 Million) | -1.9%  (-1.9 Million) | -2.1%  (-2.2 Million) |
|  | Optimal | -9.3%  (-40.0 Million) | -12.4%  (-59.0 Million) | -3.4%  (-3.4 Million) | -3.8%  (-4.0 Million) |

Source: Authors

Table S 11 Average annual changes in GHG emission by country and ranked by descending order. Changes are calculated with respect to the BAU scenario and therefore negative value indicate a reduction in emissions. Data refers to the average CSA-tailoring scenario.

| **Rule 1: Adoption based on yield increases** | | | **Rule 2: Adoption based on reduction of emission intensity and yield increases** | | |
| --- | --- | --- | --- | --- | --- |
|  | **Climate Model** | |  | **Climate Model** | |
| Country | **GFDL**  (Mg CO_2_ e yr^-1^) | **HADGEM**  (Mg CO_2_ e yr^-1^) | **Country** | **GFDL**  (Mg CO_2_ e yr^-1^) | **HADGEM**  (Mg CO_2_ e yr^-1^) |
| USA | 4,916,224 | 9,711,588 | ETH | 201,104 | 326,144 |
| ETH | 2,883,631 | 2,825,533 | UGA | 165,427 | 202,878 |
| RUS | 2,196,371 | 9,408,867 | CMR | 72,885 | 72,543 |
| KAZ | 684,457 | 1,020,043 | COD | 27,718 | 4,772 |
| UKR | 629,222 | 446,631 | GAB | 2,319 | 2,496 |
| ROU | 539,499 | 630,106 | COG | 346 | 483 |
| CAN | 487,123 | 48,026 | NER | 0 | -105 |
| PAK | 439,858 | 419,157 | SWE | 0 | -8,588 |
| MEX | 322,432 | 406,880 | SWZ | -2 | 396 |
| JPN | 294,761 | 208,982 | KWT | -4 | -11 |
| KOR | 272,001 | 172,671 | PNG | -75 | -167 |
| TUR | 251,876 | 19,163 | PRI | -97 | -253 |
| SRB | 221,758 | 292,100 | LTU | -282 | -92 |
| UGA | 218,472 | 206,603 | CYP | -923 | -1,275 |
| IRN | 158,980 | 82,427 | NAM | -1,630 | -1,524 |
| BGR | 158,774 | 131,799 | BLR | -1,841 | -972 |
| LAO | 105,725 | -30,487 | YEM | -1,999 | -598 |
| CMR | 90,422 | 105,849 | BTN | -2,009 | -3,068 |
| AZE | 78,008 | 63,456 | GEO | -2,338 | -874 |
| HUN | 53,917 | 295,141 | HND | -2,769 | -2,439 |
| COD | 53,370 | 20,130 | MNG | -2,809 | -2,821 |
| SWE | 49,633 | 62,376 | NZL | -3,432 | -3,205 |
| MDA | 35,577 | 32,375 | SVN | -3,574 | -3,671 |
| SWZ | 32,537 | 39,490 | SOM | -3,621 | -3,362 |
| CHL | 30,305 | 20,335 | BLZ | -5,240 | -6,915 |
| SVK | 22,324 | 29,664 | ERI | -5,268 | -2,928 |
| AFG | 17,842 | -74,268 | CAF | -5,632 | -5,881 |
| FIN | 9,318 | 10,606 | LBY | -6,123 | -5,005 |
| ERI | 7,879 | -101 | ALB | -6,183 | -5,861 |
| MNG | 5,785 | 7,328 | KGZ | -7,453 | -4,731 |
| BLR | 4,382 | 6,208 | BDI | -7,456 | -11,034 |
| GAB | 2,825 | 3,042 | GTM | -7,759 | -7,393 |
| BDI | 1,912 | -4,624 | URY | -8,493 | -10,968 |
| LBY | 975 | 1,255 | TJK | -9,050 | -53,162 |
| YEM | 532 | 3,519 | FIN | -10,008 | -14,956 |
| COG | 459 | 610 | CHL | -10,403 | -11,298 |
| SDN | 121 | 5,565 | TCD | -10,886 | -8,536 |
| KWT | 22 | 53 | MDA | -14,234 | -43,882 |
| LTU | -88 | 163 | MRT | -15,139 | -7,546 |
| PNG | -112 | -163 | CZE | -15,781 | -5,092 |
| NER | -130 | -4 | PRT | -16,142 | -12,090 |
| PRI | -220 | -196 | JOR | -16,596 | -30,563 |
| CAF | -329 | -542 | KOR | -16,644 | -18,488 |
| CYP | -907 | -1,207 | POL | -18,533 | -24,554 |
| NAM | -1,144 | -733 | PAN | -18,582 | -2,081 |
| BTN | -1,992 | -2,828 | GNB | -19,359 | -23,019 |
| KGZ | -2,298 | -1,046 | TLS | -20,764 | -44,281 |
| BLZ | -2,380 | -4,429 | SVK | -20,803 | -37,674 |
| SVN | -2,417 | 1,417 | SAU | -21,019 | -29,938 |
| HND | -2,462 | -8,298 | NLD | -21,712 | -18,921 |
| GEO | -2,778 | 11,601 | CRI | -22,866 | -28,314 |
| SOM | -2,927 | -2,652 | JPN | -24,032 | -56,561 |
| NZL | -3,337 | -3,309 | SLV | -27,004 | -37,649 |
| IRQ | -4,613 | -17,168 | NIC | -27,053 | -16,398 |
| ALB | -5,344 | -4,946 | AZE | -28,526 | -33,350 |
| CZE | -6,949 | -8,050 | SDN | -28,608 | -18,175 |
| GUY | -7,429 | -33,152 | BEN | -29,713 | -31,414 |
| URY | -8,049 | -10,339 | TUN | -36,781 | -16,654 |
| TJK | -12,433 | -45,267 | AUS | -39,535 | -61,147 |
| TCD | -13,503 | -4,387 | SLE | -40,152 | -53,340 |
| ECU | -13,613 | -116,182 | CIV | -42,173 | -42,591 |
| PRT | -14,415 | -11,398 | DOM | -45,596 | -52,991 |
| MRT | -15,055 | -7,508 | TGO | -47,232 | -45,089 |
| NIC | -15,890 | -22,514 | GHA | -47,388 | -56,287 |
| PAN | -16,871 | -5,830 | AUT | -48,829 | -51,511 |
| TLS | -17,379 | -46,285 | GUY | -48,949 | -75,317 |
| NLD | -19,064 | -17,347 | BEL | -49,642 | -47,692 |
| GNB | -19,547 | -21,906 | LAO | -57,064 | -44,510 |
| DOM | -21,115 | -26,515 | CUB | -58,542 | -80,488 |
| JOR | -21,295 | -32,216 | BGR | -70,555 | -70,536 |
| KEN | -22,259 | 43,282 | ROU | -72,024 | -65,527 |
| CRI | -22,939 | -27,801 | BFA | -72,058 | -51,459 |
| UZB | -24,787 | -72,209 | MYS | -74,170 | -68,848 |
| SAU | -26,655 | 9,968 | UZB | -74,651 | -145,840 |
| GHA | -26,971 | -28,752 | SRB | -78,803 | -85,667 |
| CUB | -26,982 | -35,804 | KEN | -80,251 | -3,574 |
| AUS | -27,450 | -32,906 | ARG | -84,120 | -128,866 |
| TUN | -29,207 | -31,064 | HRV | -84,254 | -53,515 |
| BEN | -30,109 | -31,818 | GRC | -86,275 | -78,637 |
| CIV | -34,674 | -24,458 | PRY | -86,517 | -106,021 |
| SLE | -37,411 | -46,838 | SEN | -100,581 | -105,293 |
| PRY | -41,576 | -84,897 | ECU | -102,096 | -165,769 |
| TGO | -46,820 | -44,069 | ZMB | -123,117 | -91,596 |
| BEL | -51,428 | -44,715 | MOZ | -127,567 | -140,631 |
| AUT | -51,637 | -44,925 | KHM | -127,576 | -263,377 |
| SLV | -56,327 | -34,160 | COL | -134,484 | -200,689 |
| MYS | -57,304 | -51,930 | MAR | -143,032 | -120,141 |
| TKM | -57,492 | 34 | AGO | -145,082 | 40,115 |
| GRC | -59,552 | -64,245 | PRK | -151,350 | -165,251 |
| BFA | -61,443 | -50,533 | MWI | -151,844 | -134,912 |
| POL | -62,111 | 46,686 | TKM | -152,538 | -101,276 |
| ARG | -66,421 | -110,611 | LKA | -159,079 | -48,054 |
| HRV | -71,402 | -20,247 | AFG | -161,525 | -229,081 |
| AGO | -79,706 | 57,821 | DZA | -171,781 | -195,978 |
| SEN | -81,865 | -89,686 | DEU | -187,735 | -216,586 |
| COL | -89,176 | -128,648 | IRQ | -190,904 | -202,814 |
| BOL | -97,524 | 37,266 | ESP | -202,320 | -197,447 |
| ITA | -100,711 | -23,060 | SYR | -205,915 | -278,762 |
| ZMB | -101,394 | -83,801 | HUN | -257,133 | -336,126 |
| KHM | -104,142 | -238,135 | BOL | -271,744 | -189,915 |
| MWI | -107,446 | -98,255 | GIN | -286,479 | -297,736 |
| MOZ | -121,895 | -124,354 | PER | -300,830 | -118,665 |
| MAR | -125,375 | -127,117 | GBR | -313,225 | -304,744 |
| PER | -131,701 | -23,636 | MLI | -355,388 | -279,790 |
| DZA | -134,850 | -162,492 | ZWE | -365,804 | -344,423 |
| LKA | -137,368 | -65,691 | NGA | -403,548 | -387,292 |
| PRK | -139,015 | -155,881 | ITA | -458,246 | -547,516 |
| DEU | -144,030 | -135,622 | EGY | -472,567 | -305,605 |
| ESP | -145,910 | -91,742 | VEN | -506,485 | -461,601 |
| SYR | -195,288 | -223,588 | TUR | -612,497 | -573,776 |
| MLI | -221,451 | -138,767 | TZA | -613,227 | -631,080 |
| GBR | -266,026 | -151,615 | ZAF | -705,239 | -675,158 |
| GIN | -271,211 | -268,445 | CAN | -722,463 | -464,730 |
| NGA | -273,976 | -248,988 | UKR | -801,014 | -615,116 |
| GTM | -291,911 | -111,081 | KAZ | -836,502 | -490,702 |
| BRA | -304,080 | -869,721 | FRA | -860,648 | -907,372 |
| ZWE | -323,633 | -320,430 | IRN | -907,685 | -837,807 |
| ZAF | -390,477 | -552,565 | BRA | -1,055,802 | -1,681,104 |
| VEN | -435,470 | -366,210 | MMR | -1,183,842 | -1,104,379 |
| FRA | -531,474 | -351,730 | PAK | -1,614,068 | -962,532 |
| TZA | -551,488 | -511,569 | MEX | -1,925,425 | -2,228,600 |
| NPL | -594,395 | -1,169,538 | MDG | -2,359,416 | -2,242,408 |
| EGY | -618,592 | -352,610 | PHL | -2,529,014 | -2,706,404 |
| MMR | -756,897 | -721,434 | THA | -2,631,945 | -2,104,965 |
| THA | -1,745,083 | -1,514,832 | NPL | -2,900,360 | -2,720,109 |
| PHL | -2,101,457 | -2,175,319 | RUS | -3,617,426 | -1,422,063 |
| MDG | -2,118,280 | -2,090,113 | USA | -3,787,794 | -3,972,389 |
| VNM | -3,279,705 | -3,847,205 | VNM | -3,982,757 | -4,141,204 |
| CHN | -4,295,313 | -4,012,764 | IDN | -5,742,316 | -5,800,871 |
| IDN | -4,763,782 | -4,941,183 | BGD | -7,161,971 | -5,785,394 |
| BGD | -6,284,337 | -4,492,161 | CHN | -10,973,661 | -11,098,721 |
| IND | -31,844,693 | -32,032,043 | IND | -37,033,156 | -37,710,495 |
| ***TOTALS*** | ***-50,270,863*** | ***-37,514,099*** |  | ***-102,794,432*** | ***-98,846,406*** |

Source: Authors

Table S 12 Percent change in harvested area in 2050 compared to BAU under different climate and adoption scenarios.

| Scenario | CSA Tailoring | Maize  GFDL – HADGEM | | Wheat  GFDL – HADGEM | | | Rice  GFDL – HADGEM | | |
| --- | --- | --- | --- | --- | --- | --- | --- | --- | --- |
| Rule 1: Adoption of CSA practices dependent on increased yields | Lower | 0.3% | 0.1% | | -0.5% | -0.8% | | -2.1% | -2.4% |
|  | Average | 0.7% | 0.06% | | -1.7% | -2.4% | | -5% | -5.1% |
|  | Optimal | 0.3% | -0.6% | | -3.7% | -5.4% | | -9.0% | -8.9% |
| Rule 2: Adoption of CSA practices dependent on reduction of emission intensity *and* increased yields | Lower | 0.6% | 0.4% | | -0.4% | -0.7% | | -2.4% | -2.5% |
|  | Average | 1.1% | 0.6% | | -1.1% | -1.8% | | -4.9% | -5.0% |
|  | Optimal | 1.5% | 0.6% | | -2.6% | -4.1% | | -8.5% | -8.6% |

Source: Authors

Table S 13 Largest increase in cropland areas due to reduction in areas allocated to maize, wheat and rice. Data refers to the average CSA-tailoring scenario

|  | Rule 1: Adoption of CSA practices dependent on increased yields | Rule 2: Adoption of CSA practices dependent on reduction of emission intensity and increased yields |
| --- | --- | --- |
| Crop | Increase in area (Hectares) | Increase in area (Hectares) |
| Soybean | 834,000 | 729,000 |
| Vegetables | 698,000 | 620,000 |
| Minor crops^[[2]](#footnote-2)^ | 254,000 | 213,000 |
| Temperate Fruit | 217,000 | 202,000 |
| Sugarcane | 178,000 | 192,000 |

Source: Authors

Table S 14 Projected changes in the number of animals and emissions under different climate and adoption scenarios in the year 2050.

|  | CSA Tailoring | Climate model | | | | | | |
| --- | --- | --- | --- | --- | --- | --- | --- | --- |
|  |  | GFDL | | | HADGEM | | | |
|  |  | Change in beef animals  (# heads) | Change in dairy animals  (# heads) | Additional emissions in 2050  (Mt CO_2_ e) | Change in beef animals  (# heads) | | Change in dairy animals  (# heads) | Additional emissions in 2050  (Mt CO_2_ e) |
| Rule 1: Adoption of CSA practices dependent on increased yields | Lower | 1,003,200 | 667,000 | 1.4 | | 1,460,800 | 873,000 | 2.0 |
|  | Average | 3,405,600 | 2,106,000 | 4.7 | | 4,562,800 | 2,667,000 | 6.2 |
|  | Optimal | 7,510,800 | 4,511,000 | 10.3 | | 9,746,000 | 5,536,000 | 13.1 |
| Rule 2: Adoption of CSA practices dependent on reduction of emission intensity and increased yields | Lower | 827,200 | 610,000 | 1.2 | | 1,262,800 | 809,000 | 1.8 |
|  | Average | 2,420,000 | 1,476,000 | 3.3 | | 3,454,000 | 1,985,000 | 4.7 |
|  | Optimal | 5,205,200 | 2,881,000 | 7.0 | | 7,207,200 | 3,889,000 | 9.6 |

Source: Authors

Note: additional emissions from both animals for meat and dairy animals

Table S 15. Yearly change in area from BAU. Sum across the three crops (thousand hectares)

| Scenario | CSA Tailoring | Climate | |
| --- | --- | --- | --- |
|  |  | GFDL | HadGEM |
| Rule 1 | lower | -3,656 | -4,528 |
|  | average | -9,522 | -11,043 |
|  | optimal | -19,463 | -21,950 |
| Rule 2 | lower | -3,476 | -4,109 |
|  | average | -7,660 | -9,079 |
|  | optimal | -14,594 | -17,415 |
| Rule2+AWD | lower | -3,384 | -4,010 |
|  | average | -7,659 | -9,073 |
|  | optimal | -14,698 | -17,519 |
| Rosegrant et al. adopt. rates | lower | -2,181 | -2,730 |
|  | average | -5,232 | -6,173 |
|  | optimal | -10,700 | -12,232 |

Source: Authors

**References**

1. Jones, J. W. *et al.* The DSSAT Cropping System Model. *Eur. J. Agron.* **18**, 235–265 (2003).

2. Singh, U. & Thornton, P. K. Using Crop Models for Sustainability and Environmental-Quality Assessment. *Outlook Agric.* **21**, 209–218 (1992).

3. Aggarwal, P. K. *et al.* Analysis of Yield Trends of the Rice-Wheat System in North-Western India. *Outlook Agric.* **29**, 259–268 (2000).

4. Subash, N. & Mohan, H. S. R. Evaluation of the Impact of Climatic Trends and Variability in Rice-Wheat System Productivity Using Cropping System Model DSSAT Over the Indo-Gangetic Plains of India. *Agric. For. Meteorol.* **164**, 71–81 (2012).

5. Nelson, G. C. *et al.* *Food Security, Farming, and Climate Change to 2050: scenarios, results, policy options*. *Research Monograph* (2010). doi:10.1111/an.1995.36.5.41.

6. Rosegrant, M. W. *et al.* *Food Security in a World of Natural Resource Scarcity - The Role of Agricultural Technologies*. (International Food Policy Research Institute (IFPRI), 2014).

7. You, L., Wood, S., Wood-Sichra, U. & Wu, W. Generating global crop distribution maps: From census to grid. *Agric. Syst.* **127**, 53–60 (2014).

8. Wood-Sichra, U., Joglekar, A. B. & You, L. *Spatial Production Allocation Model (SPAM) 2005: Technical Documentation*. (2016).

9. Muller, C. & Robertson, R. D. Projecting future crop productivity for global economic modeling. *Agric. Econ.* **45**, 37–50 (2014).

10. Dellink, R., Chateau, J., Lanzi, E. & Magné, B. Long-term economic growth projections in the Shared Socioeconomic Pathways. *Glob. Environ. Chang.* **42**, 200–214 (2017).

11. IIASA. SSP Database. (2013).

12. Robinson, S. *et al.* *The International Model for Policy Analysis of Agricultural Commodities and Trade (IMPACT): Model description for version 3*. vol. 1483 (2015).

13. Springmann, M. *et al.* Global and regional health effects of future food production under climate change: A modelling study. *The Lancet* (2016) doi:10.1016/S0140-6736(15)01156-3.

14. Hachigonta, S., Nelson, G. C., Thomas, T. S. & Sibanda, L. M. Southern African Agriculture and Climate Change: A Comprehensive Analysis. (2013).

15. Jalloh, A., Nelson, G. C., Thomas, T. S., Zougmoré, R. & Roy-Macauley, H. West African Agriculture and Climate Change: A Comprehensive Analysis. (2013).

16. Sulser, T. B., Nestorova, B., Rosegrant, M. W. & van Rheenen, T. The future role of agriculture in the Arab region’s food security. *Food Secur.* **3**, S23–S48 (2011).

17. Waithaka, M., Nelson, G. C., Thomas, T. S. & Kyotalimye, M. East African Agriculture and Climate Change: A Comprehensive Analysis. (2013).

18. Nelson, G. C. *et al.* Agriculture and climate change in global scenarios: Why don’t the models agree. *Agric. Econ. (United Kingdom)* **45**, 85–101 (2014).

19. Wiebe, K. D. *et al.* Climate change impacts on agriculture in 2050 under a range of plausible socioeconomic and emissions scenarios. *Environ. Res. Lett.* **10**, 85010 (2015).

20. Rosegrant, M. W. Biofuels and Grain Prices: Impacts and Policy Responses. (2008).

21. De Pinto, A. *et al.* Low Emission Development Strategies in Agriculture. An Agriculture, Forestry, and Other Land Uses (AFOLU) Perspective. *World Dev.* **87**, 180–203 (2016).

22. Leys, C., Ley, C., Klein, O., Bernard, P. & Licata, L. Detecting outliers: Do not use standard deviation around the mean, use absolute deviation around the median. *J. Exp. Soc. Psychol.* **49**, 764–766 (2013).

23. Reicosky, D. C., Lindstrom, M. J., Schumacher, T. E., Lobb, D. A. & Malo, D. D. Tillage-induced CO2 loss across an eroded landscape. *Soil Tillage Res.* **81**, 183–194 (2005).

24. Hobbs, P. R., Sayre, K. & Gupta, R. The Role of Conservation Agriculture in Sustainable Agriculture. *Philos. Trans. R. Soc. B-Biological Sci.* **363**, 543–555 (2008).

25. Thierfelder, C., Mwila, M. & Rusinamhodzi, L. Conservation agriculture in eastern and southern provinces of Zambia: Long-term effects on soil quality and maize productivity. *Soil Tillage Res.* **126**, 246–258 (2013).

26. Vanlauwe, B. *et al.* Agronomic Use Efficiency of N Fertilizer in Maize-based Systems in Sub-Saharan Africa within the Context of Integrated Soil Fertility Management. *Plant Soil* **339**, 35–50 (2011).

27. Smith, P. *et al.* Agriculture, Forestry and Other Land Use (AFOLU). *Clim. Chang. 2014 Mitig. Clim. Chang. Contrib. Work. Gr. III to Fifth Assess. Rep. Intergov. Panel Clim. Chang. [Edenhofer, O., R. Pichs-Madruga, Y. Sokona, E. Farahani, S. Kadner, K. Seyboth, A. Adler,* 811–922 (2014) doi:10.1016/j.phrs.2011.03.002.

28. FAO. Module 1: Why Climate-Smart Agriculture, Forestry and Fisheries. in *Climate Smart Agriculture Sourcebook* (Food and Agriculture Organization of the United Nations (FAO), 2013).

29. Tyagi, L., Kumari, B. & Singh, S. N. Water management - A tool for methane mitigation from irrigated paddy fields. *Sci. Total Environ.* **408**, 1085–1090 (2010).

30. Ainsworth, E. A. & Long, S. P. What have we learned from 15 years of free-air CO2 enrichment (FACE)? A meta-analytic review of the responses of photosynthesis, canopy properties and plant production to rising CO2. *New Phytol.* **165**, 351–372 (2005).

31. Ainsworth, E. A. & Ort, D. R. How Do We Improve Crop Production in a Warming World? *Plant Physiol.* **154**, 526–530 (2010).

32. Sharkey, T. D., Bernacchi, C. J., Farquhar, G. D. & Singsaas, E. L. Fitting photosynthetic carbon dioxide response curves for C3 leaves. *Plant, Cell Environ.* **30**, 1035–40 (2007).

33. Asseng, S. *et al.* Climate change impact and adaptation for wheat protein. *Glob. Chang. Biol.* **25**, 155–173 (2019).

34. Ruane, A. *et al.* Biophysical and economic implications for agriculture of +1.5° and +2.0°C global warming using AgMIP Coordinated Global and Regional Assessments. *Clim. Res.* **76**, 17–39 (2018).

35. Jones, A. G., Scullion, J., Ostle, N., Levy, P. E. & Gwynn-Jones, D. Completing the FACE of elevated CO2 research. *Environ. Int.* **73**, 252–258 (2014).

36. Hasegawa, T. *et al.* Risk of increased food insecurity under stringent global climate change mitigation policy. *Nat. Clim. Chang.* **8**, 699–703 (2018).

37. Schleussner, C. F. *et al.* Crop productivity changes in 1.5 °c and 2 °c worlds under climate sensitivity uncertainty. *Environ. Res. Lett.* **13**, (2018).

38. Deryng, D. *et al.* Regional disparities in the beneficial effects of rising CO 2 concentrations on crop water productivity. *Nat. Clim. Chang.* **6**, 786–790 (2016).

39. Boote, K. J., Allen Jr., L. H., Vara Prasad, P. V. & Jones, J. W. Testing effects of climate change in crop models. in *Handbook of Climate Change and Agroecosystems* (eds. Hillel, D. & Rosenzweig, C.) 109–157 (2011).

40. Prentice, C. *et al.* The carbon cycle and atmospheric carbon dioxide. in *Climate Change 2001: The Scientific Basis. Contributions of working group I to the third assessment report of the intergovernmental panel on climate change* (ed. . J. T. Houghton, Y. Ding, D. J. Griggs, M. Noguer, P. J. Van der Linder, X. Dai, K. M. & C. A. J.) (Cambridge, UK: Cambridge University Press., 2001). doi:10.1256/004316502320517344.

41. Pritchard, S. G. & Amthor, J. S. *Crops and environmental change: an introduction to effects of global warming, increasing atmospheric CO2 and O3 concentrations, and soil salinization on crop physiology and yield*. (New York: Food Products Press, 2005).

42. Leakey, A. D. B. *et al.* Elevated CO2 effects on plant carbon, nitrogen, and water relations: six important lessons from FACE. *J. Exp. Bot.* **60**, 2859–2876 (2009).

43. Zavala, J. A., Casteel, C. L., DeLucia, E. H. & Berenbaum, M. R. Anthropogenic increase in carbon dioxide compromises plant defense against invasive insects. *Proc. Natl. Acad. Sci.* **105**, 5129 LP – 5133 (2008).

44. Farooq, U., Iqbal, M. & Bashir, A. Cost and Revenue Statistics of Paddy Production: Farmer’s Perspective. *Int. J. Agric. Biol.* **1**, (1999).

45. Greer, C. A. *et al.* *Sample costs to produce rice*. http://cecolusa.ucdavis.edu/files/153082.pdf (2012).

46. Perret, S.-R., Saringkarn, P., Jourdain, D. & Babel, M. S. Can rice farmers pay irrigation costs? An investigation of irrigation supply costs and use value in a case study scheme in Thailand. *Cah. Agric.* **22**, 385–392 (2013).

47. Kürschner, E. *et al.* *SLE Publication Series – S241 Water Saving in Rice Production– Dissemination, Adoption and Short Term Impacts of Alternate Wetting and Drying (AWD) in Bangladesh*. *SLE Publication Series* http://edoc.hu-berlin.de/series/sle/241/PDF/241.pdf (2010).

48. Lampayan, R. M. Water Saving Work Group. Development, dissemination, and adoption of technologies to help farmers cope with water scarcity. (2012).

49. Gerber, P. J. *et al.* Technical options for the mitigation of direct methane and nitrous oxide emissions from livestock: a review. *Animal : an international journal of animal bioscience* vol. 7 Suppl 2 220–234 (2013).

50. Cenacchi, N. *et al.* Climate change, agriculture, and adaptation in the Republic of Korea to 2050: An integrated assessment. (2016).

51. Huda, A. *et al.* Floodwater ammonium, nitrogen use efficiency and rice yields with fertilizer deep placement and alternate wetting and drying under triple rice cropping systems. *Nutr. Cycl. Agroecosystems* **104**, 53–66 (2016).

52. Pandey, A. *et al.* Organic matter and water management strategies to reduce methane and nitrous oxide emissions from rice paddies in Vietnam. *Agric. Ecosyst. Environ.* **196**, 137–146 (2014).

53. Chivenge, P. *et al.* Organic and Mineral Input Management to Enhance Crop Productivity in Central Kenya. *Agron. J.* **101**, 1266–1275 (2009).

54. Agegnehu, G., Vanbeek, C. & Bird, M. I. Influence of integrated soil fertility management in wheat and tef productivity and soil chemical properties in the highland tropical environment. *J. Soil Sci. Plant Nutr.* **14**, 532–545 (2014).

55. Erenstein, O. *Zero Tillage in the Rice-Wheat Systems of the Indo-Gangetic Plains A Review of Impacts and Sustainability Implications*. vol. 00916 (2009).

56. Govaerts, B., Sayre, K. D. & Deckers, J. Stable High Yields with Zero Tillage and Permanent Bed Planting? *F. Crop. Res.* **94**, 33–42 (2005).

57. Ito, M., Matsumoto, T. & Quinones, M. A. Conservation Tillage Practice in sub-Saharan Africa: The Experience of Sasakawa Global 2000. *Crop Prot.* **26**, 417–423 (2007).

58. Bandaogo, A. *et al.* Effect of fertilizer deep placement with urea supergranule on nitrogen use efficiency of irrigated rice in Sourou Valley (Burkina Faso). *Nutr. Cycl. Agroecosystems* **102**, 79–89 (2015).

59. Powlson, D. S. *et al.* Limited potential of no-till agriculture for climate change mitigation. *Nat. Clim. Chang.* **4**, 678–683 (2014).

60. Denef, K., Archibeque, S. & Paustian, K. *Greenhouse Gas Emissions from U . S . Agriculture and Forestry : A Review of Emission Sources , Controlling Factors , and Mitigation Potential*. *Interim report to USDA under Contract #GS23F8182H* (2011).

61. Ogle, S. M., Breidt, F. J. & Paustian, K. Agricultural Management Impacts on Soil Organic Carbon Storage Under Moist and Dry Climatic Conditions of Temperate and Tropical Regions. *Biogeochemistry* **72**, 87–121 (2005).

62. Ogle, S. M., Archibeque, S. L., Gurung, R. & Paustian, K. *Report on GHG Mitigation Literature Review for Agricultural Systems*. (2010).

63. Six, J. *et al.* The Potential to Mitigate Global Warming with No-Tillage Management is only Realized when Practised in the Long Term. *Glob. Chang. Biol.* **10**, 155–160 (2004).

64. West, T. O. & Marland, G. A synthesis of carbon sequestration, carbon emissions, and net carbon flux in agriculture: comparing tillage practices in the United States. *Agric. Ecosyst. Environ.* **91**, 217–232 (2002).

65. Beheydt, D., Boeckx, P., Ahmed, H. P. & Van Cleemput, O. N(2)O emission from conventional and minimum-tilled soils. *Biol. Fertil. Soils* **44**, 863–873 (2008).

66. Wang, M. *et al.* Sources of methane in China: rice fields, agricultural waste treatment, cattle, coal mines, and other minor sources. *Sci. Atmos. Sin.* **17**, 52–64 (1993).

67. Wang, M. X. Methane Emission Rates from Various Rice Fields in China. in *China Contribution to Global Change Studies* (eds. Zheng, Y. Du & Hai, L.) 154–160 (Science Press, 1995).

68. Lu, W., Chen, W., Guo, W. & Duan, B. Emission of Methane from the Rice Field Affected by Irrigation. in *The Planning Meeting of Methane Emission from Rice Field* 19–26 (China National Rice Reserach Institute, 1995).

69. Adhya, T. K. *et al.* Methane emission from flooded rice fields under irrigated conditions. *Biol. Fertil. Soils* **18**, 245–248 (1994).

70. Nugroho, S. G. *et al.* Effect of intermittent irrigation on methane emission from an indonesian paddy field. *Soil Sci. Plant Nutr.* **40**, 609–615 (1994).

71. Holzapfel-Pschorn, A. & Seiler, W. Methane emission during a cultivation period from an Italian rice paddy. *J. Geophys. Res.* **91**, 11803 (1986).

72. Schütz, H., Holzapfel-Pschorn, A., Conrad, R., Rennenberg, H. & Seiler, W. A 3-year continuous record on the influence of daytime, season, and fertilizer treatment on methane emission rates from an Italian rice paddy. *J. Geophys. Res.* **94**, 16405 (1989).

73. Shin, Y. K., Yun, S. H. & Park, M. E. Estimation of Methane Emission by Water Management and Rice Straw Application in Paddy Soil in Korea. *J. Korean Soc. Soil Sci. Fertil.* **28**, 261–65 (1995).

74. Metra-Corton, T., Bajita, J. B., Asisk, C. A., Pamplona, R. R. & Salamanca. *Methane emissions from an irrigated rice field*. (1995).

75. Murase, J. *et al.* Methane emission from plots with differences in fertilizer application in thai paddy fields. *Soil Sci. Plant Nutr.* **40**, 63–71 (1994).

76. Lindau, C. W., Bollich, P. K., Delaune, R. D., Patrick, W. H. & Law, V. J. Effect of Urea Fertilizer and Environmental-Factors On Ch4 Emissions From a Louisiana, Usa Rice Field . *Plant Soil*  **136**, 195–203 (1991).

77. Cicerone, R. J., Delwiche, C. C., Tyler, S. C. & Zimmerman, P. R. Methane emissions from California rice paddies with varied treatments. *Global Biogeochem. Cycles* **6**, 233–248 (1992).

78. Sass, R. L. & Fisher, F. M. Methane Emissions from Texax Rice Fields: A Five Year Study. in *Climate Change and Rice* (eds. Peng, S., Ingram, K. T., Neue, H. U. & Ziska, L. H.) 46–59 (Springer-Verlag, 1995).

79. Adhya, T. K., Linquist, B., Searchinger, T., Wassmann, R. & Yan, X. *Wetting and Drying: reducing Greenhouse Gas Emissions and Saving Water from Rice Production*. vol. Installmen (2014).

80. Cai, Z. C. *et al.* Measurements of CH4 and N2O emissions from rice paddies in Fengqiu, China. *Soil Sci. Plant Nutr.* **45**, 1–13 (1999).

81. Xiong, Z. Q. *et al.* Measurement of nitrous oxide emissions from two rice-based cropping systems in China. in *Nutrient Cycling in Agroecosystems* vol. 64 125–133 (2002).

82. Khalil, M. A. K. *et al.* Emissions of methane, nitrous oxide, and other trace gases from rice fields in China. *J. Geophys. Res. Atmos.* **103**, 25241–25250 (1998).

83. Zheng, X. *et al.* Impacts of soil moisture on nitrous oxide emission from croplands: A case study on the rice-based agro-ecosystem in Southeast China. *Chemosph. - Glob. Chang. Sci.* **2**, 207–224 (2000).

84. Kumar, U., Jain, M. C., Pathak, H., Kumar, S. & Majumdar, D. Nitrous oxide emission from different fertilizers and its mitigation by nitrification inhibitors in irrigated rice. *Biol. Fertil. Soils* **32**, 474–478 (2000).

85. Majumdar, D., Kumar, S., Pathak, H., Jain, M. . & Kumar, U. Reducing nitrous oxide emission from an irrigated rice field of North India with nitrification inhibitors. *Agric. Ecosyst. Environ.* **81**, 163–169 (2000).

86. Pathak, H. *et al.* Emission of nitrous-oxide from rice-wheat systems of indo-gangetic plains of India. *Environ. Monit. Assess.* **77**, 163–178 (2002).

87. Suratno, W. *et al.* Nitrous oxide flux from irrigated rice fields in West Java. in *Environmental Pollution* vol. 102 159–166 (1998).

88. Yagi, K., Tsuruta, H., Kanda, K. I. & Minami, K. Effect of water management on methane emission from a Japanese rice paddy field: Automated methane monitoring. *Global Biogeochem. Cycles* **10**, 255–267 (1996).

89. Bronson, K. F., Neue, H.-U., Abao, E. B. & Singh, U. Automated Chamber Measurements of Methane and Nitrous Oxide Flux in a Flooded Rice Soil: I. Residue, Nitrogen, and Water Management. *Soil Sci. Soc. Am. J.* **61**, 981 (1997).

90. Dong, N. M. *et al.* Effects of alternating wetting and drying versus continuous flooding on fertilizer nitrogen fate in rice fields in the Mekong Delta, Vietnam. *Soil Biol. Biochem.* **47**, 166–174 (2012).

91. Gaihre, Y. K. *et al.* Impacts of urea deep placement on nitrous oxide and nitric oxide emissions from rice fields in Bangladesh. *Geoderma* **259**, 370–379 (2015).

92. Muhammad, W., Vaughan, S. M., Dalal, R. C. & Menzies, N. W. Crop residues and fertilizer nitrogen influence residue decomposition and nitrous oxide emission from a Vertisol. *Biol. Fertil. Soils* **47**, 15–23 (2011).

93. Frimpong, K. A. & Baggs, E. M. Do combined applications of crop residues and inorganic fertilizer lower emission of N2O from soil? *Soil Use Manag.* **26**, 412–424 (2010).

94. Lampayan, R. M. *et al.* *Adoption of water saving technologies in rice production in the Philippines*. *Transition in Agriculture for Enhancing Water Productivity* (2003).

95. Palis, F. G. *et al.* Farmer adoption of controlled irrigation in rice: a case study in Canarem, Victoria, Tarlac. *Philipp. J. Crop Sci.* **29**, 3–12 (2004).

96. Rejesus, R. M., Palis, F. G., Rodriguez, D. G. P., Lampayan, R. M. & Bouman, B. A. M. Impact of the alternate wetting and drying (AWD) water-saving irrigation technique: Evidence from rice producers in the Philippines. *Food Policy* **36**, 280–288 (2011).

97. Lampayan, R. M., Rejesus, R. M., Singleton, G. R. & Bouman, B. A. M. Adoption and economics of alternate wetting and drying water management for irrigated lowland rice. *F. Crop. Res.* **170**, 95–108 (2015).

98. Lampayan, R. Smart Water Techniques for Rice. in (European Initiative for Agricultural Research for Development, 2013).

99. Singh, C. B., Aujla, T. S., Sandhu, B. S. & Khera, K. L. Effect of transplanting date and irrigation regime on growth, yield and water use in rice (Oryza sativa) in northern India. *Indian J. Agric. Sci.* **66**, 137–141 (1996).

100. Myhre, G. *et al.* Anthropogenic and natural radiative forcing. in *Climate Change 2013 the Physical Science Basis: Contribution of Working Group I to the Fifth Assessment Report of the Intergovernmental Panel on Climate Change* (eds. Stocker, T. F. et al.) (Cambridge University Press, 2013). doi:10.1017/CBO9781107415324.018.

101. IIASA. RCP database. https://tntcat.iiasa.ac.at/RcpDb (2015).

102. Riahi, K., Grübler, A. & Nakicenovic, N. Scenarios of long-term socio-economic and environmental development under climate stabilization. *Technol. Forecast. Soc. Change* **74**, 887–935 (2007).

103. Meinshausen, M. *et al.* The RCP Greenhouse Gas Concentrations and Their Extensions from 1765 to 2300. *Clim. Change* **109**, 213–241 (2011).

104. O’Neill, B. C. *et al.* A new scenario framework for climate change research: the concept of shared socioeconomic pathways. *Clim. Change* **122**, 387–400 (2014).

105. Dunne, J. P. *et al.* GFDL’s ESM2 Global Coupled Climate-Carbon Earth System Models. Part I: Physical Formulation and Baseline Simulation Characteristics. *J. Clim.* **25**, 6646–6665 (2012).

106. Jones, C. D. *et al.* The HadGEM2-ES Implementation of CMIP5 Centennial Simulations. *Geosci. Model Dev.* **4**, 543–570 (2011).

1. For the analysis of technology adoption (described in the following sections) we focus only on a climate change scenario based on the HadGEM climate model. See page 22 for details. [↑](#footnote-ref-1)
2. The IMPACT model considers 62 agricultural commodities, including all major cereals, oil crops, roots and tubers, meats, milk, eggs, oils, oilcakes and meals, vegetables, fruits, sugarcane and beets, and cotton. A series of crops that might be of local but not global importance are grouped in the “others” category. [↑](#footnote-ref-2)
